# An oncogenic KRAS-driven secretome involving TNFα promotes niche preparation prior to pancreatic cancer onset

**DOI:** 10.1186/s12943-025-02541-1

**Published:** 2026-02-03

**Authors:** Chantal Allgöwer, Medhanie A. Mulaw, James Nagai, Sandra Wiedenmann, Elena Anne Ringel, Benedetta Ferrara, Lorenzo Piemonti, Tengku Ibrahim Maulana, Luisa T. Ferreira, Adam Flinders, Claudia Teufel, Leon Reichardt, Paul B. Lopatta, Dharini Srinivasan, Anton Lahusen, Thomas Seufferlein, Nadine Therese Gaisa, Annika Beck, Jessica Lindenmayer, Michael K. Melzer, Eleni Zimmer, Elodie Roger, Sandra Heller, J.-Mathias Löhr, Stefan Liebau, Peter Loskill, Yuan-Na Lin, Paolo Riccardo Camisa, Claus Jorgensen, Stefano Crippa, Matthias Meier, Meike Hohwieler, Ivan G. Costa, Markus Breunig, Alexander Kleger

**Affiliations:** 1 Institute of Molecular Oncology and Stem Cell Biology, Ulm University Hospital, Albert-Einstein-Allee 23, 89081 Ulm, Germany; 2 Single Cell Sequencing Unit, Ulm University, Ulm, Germany; 3 Institute for Computational Genomics, Center for Computational Life Sciences, RWTH Aachen Medical Faculty, Aachen, Germany; 4 Helmholtz Pioneer Campus, Helmholtz Munich, Munich, Germany; 5 Diabetes Research Institute, IRCCS Ospedale San Raffaele, Milan, Italy; 6 Vita-Salute San Raffaele University, Milan, Italy; 7 Department for Microphysiological Systems, Institute of Biomedical Engineering, Faculty of Medicine, Eberhard Karls University Tübingen, Tübingen, Germany; 8 NMI Natural and Medical Sciences Institute at the University of Tübingen, Reutlingen, Germany; 9 3R Center Tübingen for In Vitro Models and Alternatives to Animal Testing, Tübingen, Germany; 10 Cancer Research UK Manchester Institute, The University of Manchester, Wilmslow Road M20 4BX, Manchester, UK; 11 Department of Internal Medicine I, Ulm University Hospital, Ulm, Germany; 12 Institute of Pathology, Ulm University Hospital, Ulm, Germany; 13 Core Facility Organoids, Ulm University, Ulm, Germany; 14 Department of Urology, Ulm University Hospital, Ulm, Germany; 15 Department of Clinical Science, Intervention and Technology, Karolinska Institute, Stockholm, Sweden; 16 Institute of Neuroanatomy & Developmental Biology, Eberhard Karls University Tübingen, Tübingen, Germany; 17 Department of Pancreatic Surgery, Pancreas Translational and Clinical Research Center, IRCCS Ospedale San Raffaele, Milan, Italy; 18 Center for Biotechnology and Biomedicine, University of Leipzig, Leipzig, Germany; 19 Division of Interdisciplinary Pancreatology, Department of Internal Medicine I, Ulm University Hospital, Ulm, Germany

## Abstract

**Background:**

Pancreatic ductal adenocarcinomas (PDACs) are highly lethal and aggressive with oncogenic KRAS being the main oncogenic driver of the disease. PDACs have been extensively profiled at advanced stages, and in advanced disease the tumor microenvironment is a major determinant that critically shapes patient outcomes. Since the molecular events occurring prior to invasive growth remain poorly understood, we aimed to investigate changes in the precancerous epithelium and its surrounding niche.

**Methods:**

We acquired time-resolved, single-cell transcriptomic (scRNAseq), and accessible-chromatin data from human pluripotent stem cell-derived pancreatic duct-like organoids (PDLO) inducibly expressing KRAS^G12D^ and from various niche cells.

**Results:**

Analysis of the pure epithelium already revealed key signatures of matrix remodeling and inflammation-related signaling upon few days of KRAS^G12D^ expression. Machine learning captured KRAS^G12D^-dependent transcriptomic classifiers with high prediction accuracy and niche preparatory relevance. Various co-culture approaches followed by scRNAseq and functional validation, including T-cell microfluidics, demonstrated that the KRAS^G12D^-induced PDLO-secretome activates pancreatic stellate cells (PaSCs) and protects precancerous organoids from T cell infiltration. Additional, *in silico* approaches reconstructed a virtual pancreatic (pre)cancerous space to profile cell–cell interactions between PDLOs and niche cells. TNFα emerged as a top-ranked ligand and was functionally validated to mediate T-cell shielding and PaSC activation. Cyst fluid from 80 prospectively sampled Intraductal Papillary Mucinous Neoplasm (IPMNs) –well-known cystic PDAC precursor lesions– showed a stepwise TNFα rise across LGD (low-grade), HGD (high-grade), and IC (invasive cancer).

**Conclusion:**

Our study reveals that oncogenic KRAS orchestrates niche-preparatory programs that precede PDAC formation and highlight a T cell exclusion program governed by epithelial-derived TNFα.

**Supplementary Information:**

The online version contains supplementary material available at 10.1186/s12943-025-02541-1.

## Introduction

Improving the prognosis of patients suffering from pancreatic ductal adenocarcinoma (PDAC) remains one of the greatest challenges in oncology with a stagnating poor overall survival rate while incidence is rising. Factors contributing to the unfavorable prognosis of PDAC include late diagnosis, a high rate of metastases, and an inherent resistance to various treatment options. Extensive multi-omics analyses have provided valuable insights into the mutational, transcriptional, proteogenomic, and epigenetic changes in PDAC but effective targeted therapies are still widely lacking [1–6]. Recent data has highlighted that specific tumor microenvironment (TME) components including various immune cells, stromal cells, and matrix factors dictate tumor behavior and treatment response [7–9]. Consequently, the TME has emerged as a compelling novel therapeutic target. However, targeting the TME requires thorough understanding of the underlying communication hubs across various cell types [10–12].

PDAC is known to traverse distinct premalignant precursor stages prior to invasive tumor growth. The most frequent precursor lesions include pancreatic intraepithelial neoplasia (PanIN), which exhibit varying degrees of dysplasia, as well as cystic neoplastic lesions either intraductal papillary mucinous neoplasm (IPMN) or mucinous cystic neoplasm (MCN) [13, 14]. Recent studies started to enhance our understanding of the molecular events at the onset of PDAC formation. However, the most extensive insights to date have been derived from rodent models [15, 16], which may not completely capture tumor progression mechanisms in humans. Recent studies employing 3D modeling techniques and spatial transcriptomics to analyze human specimens, revealed that PanINs are widespread and predominantly characterized by oncogenic KRAS mutations, thereby positioning KRAS at the pathobiological center of the earliest pancreatic (pre-)cancer stages [17–21]. While these lesions were common even in healthy tissue, their progression to PDAC appears to be restrained. Why, how, and which events finally instruct PDAC onset remains unclear but likely involve the TME [22, 23].

Although our understanding of the communication between cancer cells and the TME is steadily improving, early tumor niche preparation and programing remained largely elusive. This is partly due to the absence of effective human models that provide time-resolved insights into early stages of PDAC formation. To develop such in vitro models for studying pancreatic dysplasia and carcinogenesis, we developed protocols to differentiate pancreatic duct-like organoids (PDLOs) from both human induced pluripotent and embryonic stem cells (hiPSCs, hESCs), which display key functional characteristics of human pancreatic ducts. These PDLOs, engineered to carry specific oncogenic mutations, exhibit distinct oncogene-specific growth and molecular phenotypes, and form dysplastic lesions or PDAC-like tumors upon xenotransplantation [24–30].

Here, we employ the PDLO-KRAS^G12D^ tumor model with advanced single-cell profiling techniques and co-culture approaches to investigate early pancreatic dysplasia and tumor niche preparation at high resolution.

## Results

### A model to dissect early neoplastic states of pancreatic ductal cells

**Fig. 1:**
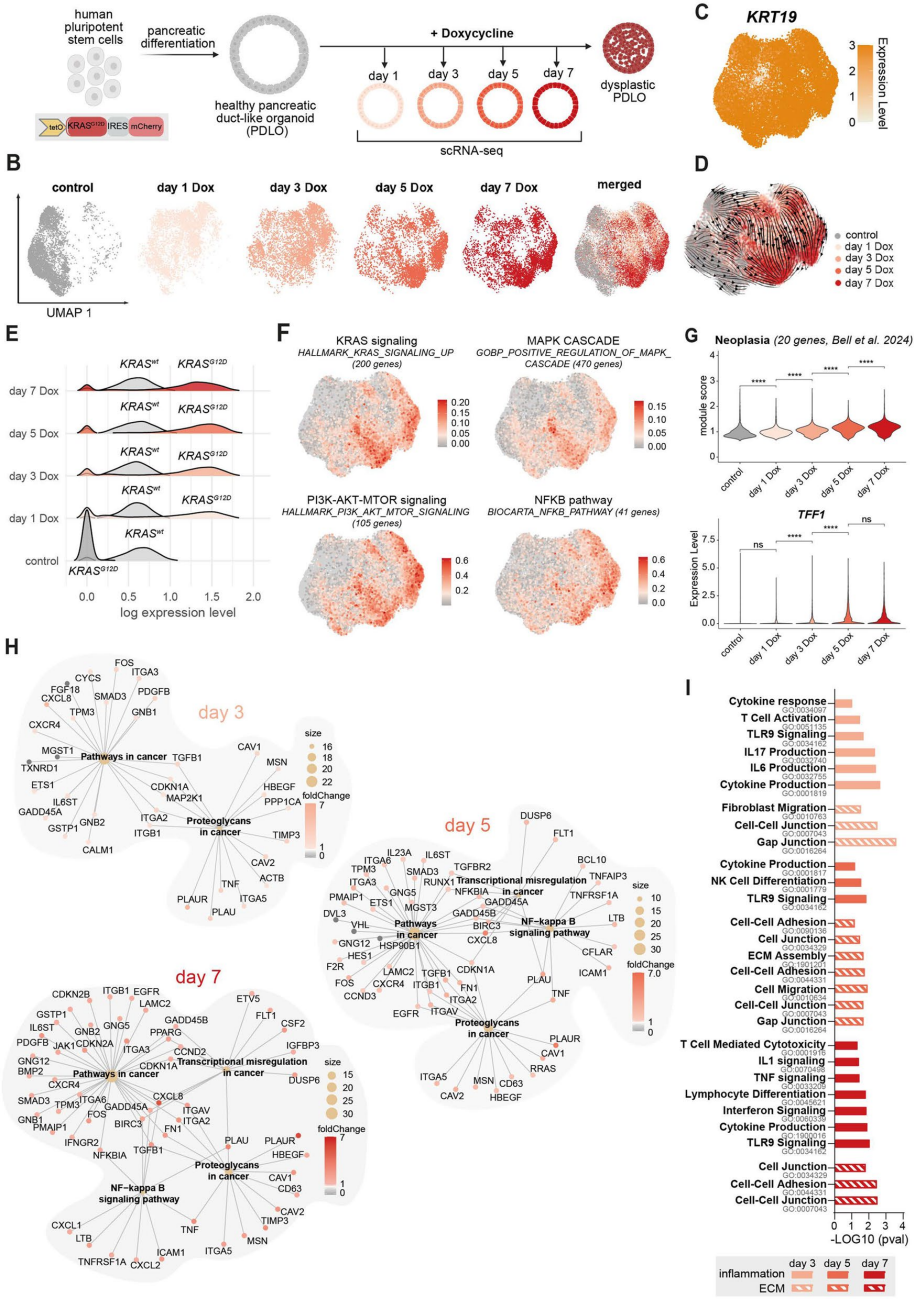
A model to dissect early neoplastic states of pancreatic ductal cells. **A** Scheme of stem cell-derived pancreatic duct-like organoid (PDLO) sampling for single-cell RNA sequencing (scRNA-seq). **B** Uniform manifold approximation and projection (UMAP) showing cells of different time points after doxycycline (Dox) treatment. **C** UMAP of KRT19 expression. **D** Velocity streamlines representing predicted trajectories within the UMAP. **E** Ridge plot showing endogenous *KRAS* (KRAS^wt^) and ectopic *KRAS*^*G12D*^ expression within different scRNA-seq samples. **F** UMAPs showing module scores of KRAS-dependent pathways. **G** Violin plots showing module score of a neoplasia signature [17] (top) and *TFF1* expression (bottom) in PDLOs over time of Dox treatment. Unpaired t-test: ****, *p* < 0.0001; ns, *p* > 0.05. **H** Gene-Concept Networks (cnet plots) illustrating KEGG pathway terms and related genes, based on transcription factor (TF) target genes. **I** Gene ontology (GO) analysis of genes derived from open chromatin regions of assay for transposase-accessible chromatin sequencing (ATAC-seq).

To chart the earliest neoplastic events of pancreatic cells, we generated time-resolved scRNA-seq data of hESC-derived PDLOs with and without KRAS^G12D^ activation (hereafter referred as KRAS-PDLOs). Transposon-based overexpression of the oncogene [25] was induced in healthy PDLOs by doxycycline (Dox) treatment, and cells were harvested for sequencing on days 1, 3, 5, and 7 (Fig. 1A-B). Robust oncogene expression was monitored by mCherry reporter signal (Supplementary Fig. 1A-B). After quality control filtering, the final scRNA-seq data set contained 23,603 cells. Almost all cells were of ductal identity (Fig. 1C, Supplementary Fig. 1 C). While a low expression of progenitor marker genes, such as *NKX6-1*, was observed, genes for non-ductal cell types such as acinar, endocrine, or endothelial cells were not expressed at relevant levels, underpinning the purity of the sequenced PDLO cultures (Supplementary Fig. 1 C).

RNA velocity analysis revealed cell trajectories aligning with the duration of Dox treatment (Fig. 1D). *KRAS*^*G12D*^ expression was stable across all time points and did not substantially affect endogenous *KRAS*^*WT*^ expression (Fig. 1E). Of note, *KRAS*^*G12D*^ engineered hESC lines carried ~ 6 KRAS copies from piggyBac transposon-based insertions of the coding sequence with a diploid native locus. PDAC cell lines or patient-derived organoids (PDO) can amplify *KRAS* to similar levels [2, 31] (Supplementary Fig. 1D), placing *KRAS*^*G12D*^ dosage in PDLOs within disease-relevant range. Immunoblots against pERK/ERK confirmed the enhanced activation of MAPK signaling along KRAS^G12D^ induction in PDLOs (Supplementary Fig. 1E). Importantly, in parental hESC-derived PDLOs, not carrying a KRAS^G12D^ cassette, Dox treatment (+ Dox) did not increase pERK/ERK ratios. Notably, ratios of Dox-treated KRAS-PDLOs ranked in the range of a heterogenous collection of PDOs and PDAC cell lines licensing our model for further application (Supplementary Fig. 1E).

To challenge our model's ability to capture molecular events driving human pre-cancer lesion formation, we assessed the activation of key tumorigenic programs. Indeed, *KRAS*^*G12D*^ expression correlated with key oncogenic programs such as MAPK and PI3K–AKT–mTOR signaling, TNFα and NF-κB signaling, epithelial–mesenchymal transition (EMT), proliferation and, senescence at all time points (Fig. 1F; Supplementary Fig. 1 F). In addition, KRAS^G12D^-PDLOs exhibited progressive expression of an early neoplasia signature and related marker genes such as *TFF1*, which were recently identified in a spatial transcriptomic analysis from low to high grade PanIN lesions [17] (Fig. 1G). Since the seemingly counteracting programs of proliferation and senescence were both upregulated after KRAS^G12D^ induction, we performed EdU-based flow cytometry and observed a selective proliferation increase of KRAS^G12D^–HA–positive cells at day 1, which was absent at later time points, in KRAS^G12D^–HA–negative cells, and in Dox-only controls (Supplementary Fig. 1G). These data support our prior observation that KRAS^G12D^-driven growth in epithelial PDLOs is rapidly curtailed by a counteracting senescence program (Supplementary Fig. 1H), with proliferation even further declining by day 9 of KRAS^G12D^ induction– while additional oncogenic events can bypass oncogene-induced senescence [25, 32]. To capture KRAS^G12D^-driven chromatin remodeling, we profiled PDLOs via ATAC-seq at days 3, 5, and 7. Already at these early timepoints, a PCA separated KRAS^G12D^ ATAC-seq samples (Supplementary Fig. 1I). Target sets of overlapping scRNA and ATAC transcription factors (Supplementary Fig. 1 J) converged on cancer-relevant signaling with prominent NF-κB pathway enrichment (Fig. 1H). Furthermore, gene-wide overrepresentation analysis of differentially opened chromatin revealed an enrichment in ECM remodeling, motility, and inflammation-related GO terms upon KRAS^G12D^ induction (F ig. 1I), indicating a rapid initiation of niche-preparatory programs and co-emergence with other key tumorigenic programs.

### KRAS^G12D^-dependent clustering of single cells reveals niche preparatory signatures

**Fig. 2:**
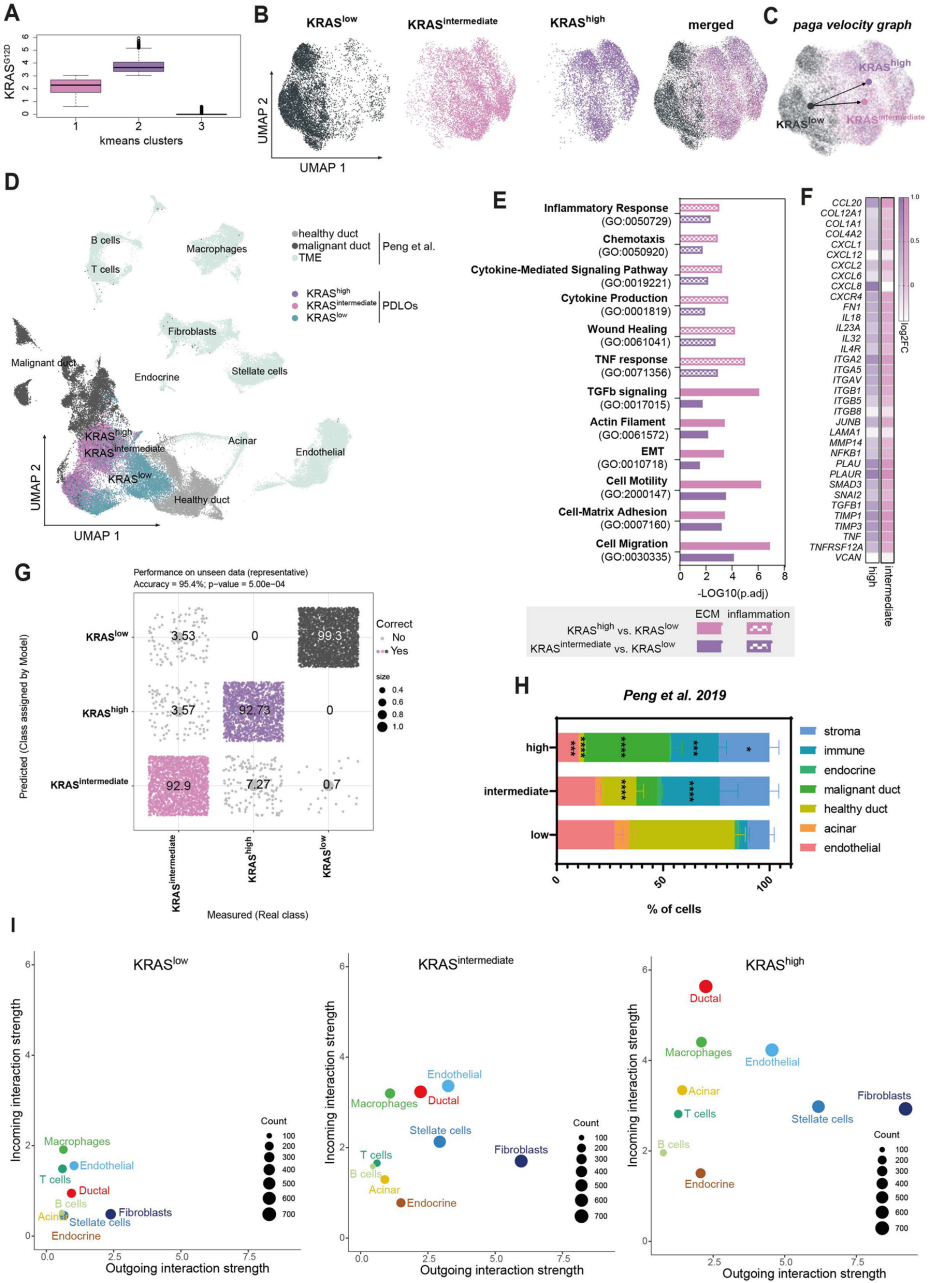
KRAS^G12D^-dependent grouping of cells reveals niche preparatory signatures. **A ***KRAS*^*G12D*^ expression of K-means clusters visualized as median with corresponding extremes. **B** UMAP illustration of K-means-based KRAS clusters. **C** Paga velocity graph showing connectivity of KRAS clusters. **D** UMAP of integrated scRNA-seq data from PDLOs and PDAC [33]. **E** GO terms related to ECM remodeling and inflammation on DEGs from KRAS^high^ and KRAS^intermediate^ cluster compared to KRAS^low^. **F** Heatmap showing log2-fold change values of selectively chosen ECM and inflammation-related genes from DEGs between KRAS^high^ and KRAS^intermediate^ clusters compared to KRAS.^low^. **G** Confusion matrix showing predicted and real classification of KRAS clusters on the validation data set (35% of the entire data set) using a deep learning algorithm. **H** Proportions and **I** interaction strength of different cell types within PDAC transcriptomes [33] when tumor samples were grouped by KRAS classifiers derived from the deep learning algorithm. Two-way ANOVA: *, *p* < 0.05; ***, *p* < 0.001; ****, *p* < 0.000533

Our polyclonal hESC KRAS^G12D^ lines featuring stochastic transposon insertions generate a mosaic of cells within the same + Dox PDLO cultures (please also see Supplementary Fig. 1 A,B). This intrinsic cell-to-cell variability enabled our scRNA-seq to resolve graded KRAS-dosage states. To account for variable transgene expression in individual cells, we grouped all cells according to their KRAS^G12D^ expression levels via K-means clustering, resulting in three clusters. Since very few reads of KRAS^G12D^ were found in 15% of cells of the lowest cluster, this cluster was defined as KRAS^low^ cluster, thereby resembling our negative control group. In contrast, all cells in the KRAS^high^ and KRAS^intermediate^ clusters expressed KRAS^G12D^ to higher levels (Fig. 2A, Supplementary Fig. 2 A). Here, KRAS^high^ and KRAS^intermediate^ UMAP clusters were well separated from the KRAS^low^ UMAP cluster (Fig. 2B). PAGA (velocity-aware) indicated distinct development of KRAS^intermediate^ and KRAS^high^ clusters, consistent with heterogeneity of KRAS^G12D^ copy numbers in the polyclonal KRAS^G12D^ bulk line. (Fig. 2C). Importantly, KRAS^low^ cells were not solely restricted to control sample input but were also found in every + Dox culture (days 1–7). To position these KRAS^G12D^-instructed cells relative to end-stage tumor cells, we integrated our PDLO scRNA-seq time-course with a published scRNA-seq data set, consisting of 24 PDAC and 11 healthy pancreas samples [33]. UMAP showed that PDLOs transcriptionally bridge healthy and malignant ductal states, progressing over time after KRAS^G12D^ induction, thus benchmarking the dataset for downstream analyses of PDAC pathogenesis. (Fig. 2D).

Although cell trajectories between KRAS^high^ and KRAS^intermediate^ clusters were distinct, no significant differentially expressed genes (DEGs) were found in the direct comparison between the two groups (KRAS^high^ vs. KRAS^intermediate^). The majority of DEGs were also shared when comparing the individual KRAS^high^ or KRAS^intermediate^ clusters against the remaining clusters (Supplementary Fig. 2B) or against the KRAS^low^ cluster (Supplementary Fig. 2 C). Unique DEGs from KRAS^high^ vs. KRAS^low^ compared to KRAS^intermediate^ vs. KRAS^low^ were more enriched for genes associated with canonical *KRAS* pathways, such as MAPK and PI3K-Akt signaling. In contrast, the unique DEGs from the comparison of KRAS^intermediate^ vs. KRAS^low^ correlated with cell–cell architecture (Supplementary Fig. 2 C). The overall high similarity indicated that functional differences between the two KRAS clusters are, besides variations in *KRAS* dosage, likely subtle. The top 50 DEGs for the KRAS^high^ and KRAS^intermediate^ clusters (vs. KRAS^low^) correlated with significantly worse overall survival in the TCGA-PAAD [4] data set (Supplementary Fig. 2D), highlighting the clinical relevance of *KRAS*^*G12D*^ signaling.

GO-term analysis of DEGs in the KRAS^high^ and KRAS^intermediate^ clusters identified significant enrichment for terms linked to classical neoplastic hallmarks and KRAS signaling, which we then grouped into self-defined, partially overlapping categories e.g. cell death, angiogenesis, metabolism, or signaling/gene-expression regulation (Supplementary Fig. 2E). Interestingly, a relatively high proportion of the enriched GO terms was associated with ECM remodeling and inflammation (Fig. 2E; Supplementary Fig. 2E), aligning with the upregulation of various cytokines, collagens, and integrins at the open chromatin, mRNA, and protein level (Fig. 1H-I; Fig. 2E-F; Supplementary Fig. 2 F). Thus, we hypothesized that KRAS^G12D^ initiates a niche-constructing program during the early stages of epithelial cell transformation, even in the absence of specific niche cell types.

To effectively capture the cellular identity driven by oncogenic KRAS in PDLOs, we implemented a deep learning algorithm based on Keras and TensorFlow. Despite the high functional similarities between the KRAS^high^ and KRAS^intermediate^ clusters, we achieved an accuracy of 95% in predicting cell identity for both clusters in our validation data set (Fig. 2G). The top 5% of predictive genes (hereafter referred to as classifiers, *n* = 1,452 genes) are displayed in Supplementary Fig. 2G. These cellular identity-inferred classifiers also allowed the accurate differentiation between healthy and tumor samples within the reanalyzed PDAC scRNA-seq data set [33], while resembling relative *KRAS*^*G12D*^ expression levels (Supplementary Fig. 2H,I). Resulting PDAC subgroups correlated with distinct morphological properties, revealing a trend towards more dedifferentiated cancers, increased perineural invasion and significantly higher levels of vascular invasion (Supplementary Fig. 2 J). Finally, grouping of the PDAC samples according to the classifiers revealed an altered composition of the TME (Fig. 2H), including an increase in stromal and immune cells, as well as enhanced cell–cell communication patterns (Fig. 2I). The classifier-dependent differences in the composition of the TME in samples of fully developed PDAC underscore the potential of oncogenic *KRAS*-instructed molecular programs to shape the TME and drive niche programming.

### KRAS^G12D^ expression induces T cell shielding and activates stellate cells

**Fig. 3:**
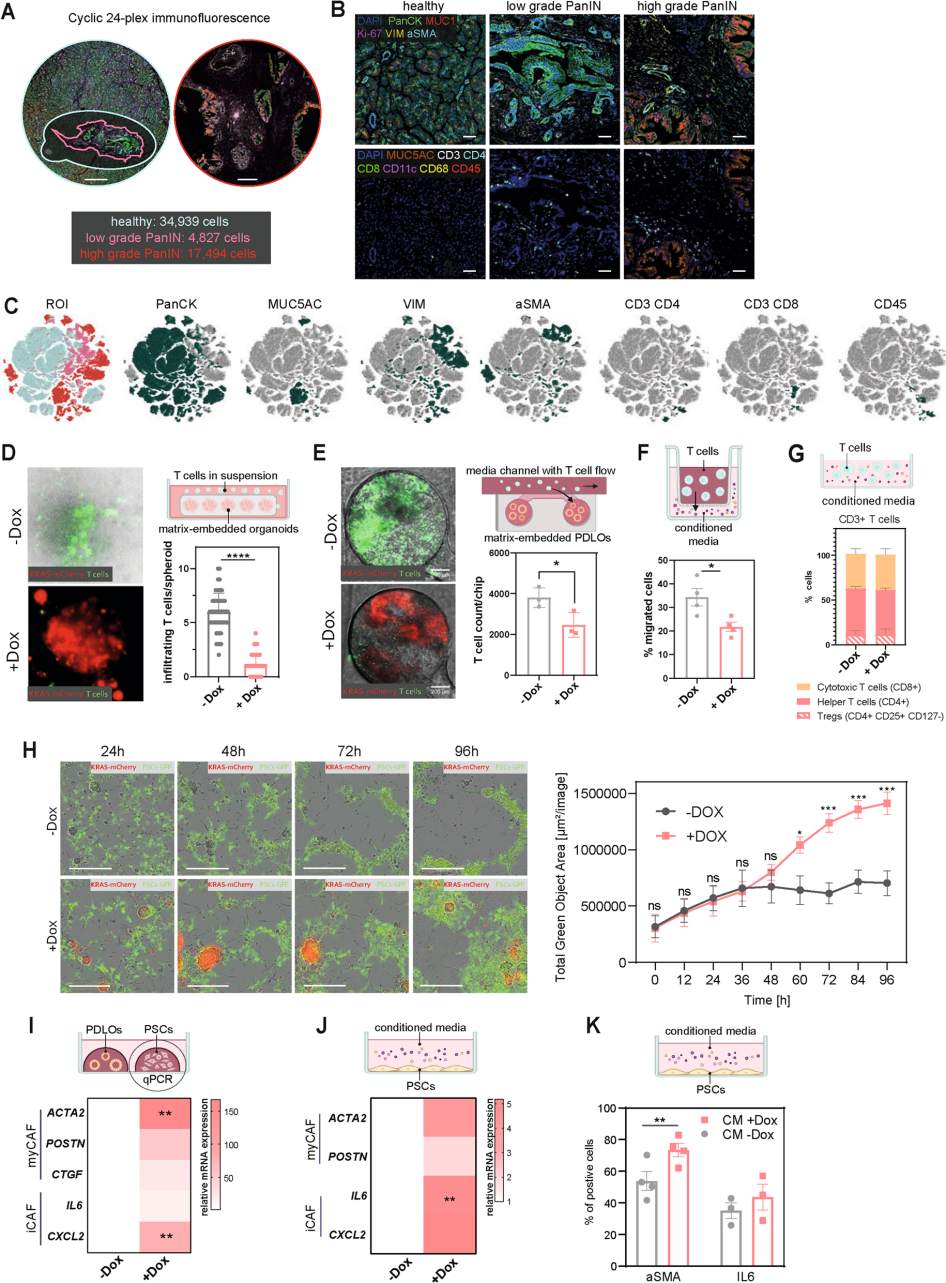
KRAS^G12D^ expression induces T cell shielding and activates stellate cells towards a CAF phenotype. **A** Overview images of human PanIN lesions of two donors, stained and imaged for 24 markers via a COMET highplex device, *n* = 1. Scale bar = 500 μm. **B** Representative image details of selected stromal (top) and immune cell markers (bottom) of human PanIN lesions subjected to 24-plex immunofluorescence staining. Scale bar = 50 μm. **C** t-SNE plots illustrating the distribution of indicated markers in human PanIN lesions from 24-plex immunofluorescence staining. **D** Representative images and respective quantification of T cells infiltrating matrix-embedded organoids after 72 h of co-culture. Mean ± standard deviation (SD) of 3 independent experiments, including a total of 5 different T cell donors. Unpaired t-test ****, *p* < 0.0001. **E** Representative images and respective quantification of T cells infiltrating matrix-embedded PDLOs within the cancer-on-chip system. Mean ± SD, *n* = 1 with 3 chips/condition. Scale bar = 200 μm. **F** Percentage of T cells migrated towards PDLO conditioned media. Mean ± standard error of mean (SEM) of 4 independent experiments. Unpaired t-test *, *p* < 0.05. **G** Proportions of the T cell subpopulations after culturing in PDLO-conditioned media for 72 h were determined by flow cytometry. Mean ± SD, *n* = 2. **H** Representative images of PDLOs co-cultured with GFP-labeled pancreatic stellate cells (PaSCs) and respective quantification of the total green area over time of live-cell imaging. Mean ± SEM, *n* = 3. Unpaired t-test ***, *p* < 0.001; ****, *p* < 0.0001. Scale bar = 400 μm. **I + J** Relative expression of CAF-related marker genes in PaSCs after indirect co-culture with KRAS^G12D^ induced PDLOs (left) or culturing in respective PDLO conditioned media for 72 h (right). The mRNA expression was normalized to *GAPDH*, and expression is shown relative to the untreated conditions (-Dox). Mean, *n* = 3, unpaired t-test**, *p* < 0.01. **K** Flow cytometry-based analysis of cells positive for αSMA and IL6 after culturing PaSCs in PDLO conditioned media (CM) for 72 h. Mean ± SEM, *n* = 4 for aSMA, *n* = 3 for IL6, unpaired t-test **, *p* < 0.01

To illustrate the niche of human PanINs in a proof-of-concept study, we employed cyclic highly multiplexed immunofluorescence of 24 markers (Fig. 3A). The increased presence of stromal components such as cancer-associated fibroblasts (CAFs) and multiple immune cells in precancer lesions supported the hypothesis of very early niche remodeling processes (Fig. 3B-C, Supplementary Fig. 3 A).

To experimentally explore the effects of epithelial *KRAS*^*G12D*^-driven paracrine signaling on major components of the niche, we established various in vitro co-culture systems. Polyclonal KRAS^G12D^ hESC-derived PDLOs were used, and Dox-treated cultures accordingly contained KRAS^low^, KRAS^intermediate^, and KRAS^high^ expression groups. Employing the previously established Interaction with Organoid-in-MatriX (InterOMaX) platform [34], we observed that significantly fewer T cells infiltrated matrix-embedded organoids expressing *KRAS*^*G12D*^ (Fig. 3D). The same shielding effect was observed when we employed a microfluidic tumor-on-chip model to evaluate recruitment of perfused T cells towards PDLOs ± KRAS^G12D^ via a porous membrane [35] (Fig. 3E). Moreover, less T cells migrated towards conditioned media (CM) derived from *KRAS*^*G12D*^-expressing PDLOs (Fig. 3F), positioning the *KRAS*^*G12D*^-instructed secretome as major driver of T-cell shielding. Importantly, the cultivation of T cells in the conditioned media did not change the composition of T cell subtypes (Fig. 3G) and had no impact on their viability, activation, or exhaustion (Supplementary Fig. 3B), indicating that the *KRAS*^*G12D*^-induced epithelial secretome primarily acted in a shielding manner. Unaltered migration towards conditioned media of parental hESC-derived PDLOs treated with dox confirmed oncogene specificity of the observed shielding phenotype (Supplementary Fig. 3 C).

Direct PDLO–PaSC co-cultures – with PaSCs as a principal CAF source [36, 37] – showed a significant PaSC growth increase within 2 days when paired with KRAS^G12D^ PDLOs (Fig. 3H). All formats used Dox-induced KRAS^G12D^ PDLOs without isolating low/intermediate/high KRAS subfractions. In contrast, PaSCs growth was not elevated in co-cultures with Dox-treated parental PDLOs (Supplementary Fig. 3D). Moreover, expression of CAF markers such as *ACTA2* and *CXCL2* was increased upon KRAS^G12D^ induction in an indirect co-culture format (Fig. 3I). A similar activation towards a CAF-like phenotype was also observed when subjecting PaSCs to conditioned medium of KRAS^G12D^-PDLOs (Fig. 3J; Supplementary Fig. 3E), which was additionally validated by flow cytometry measurements on protein level (Fig. 3K). Specifically, IL-6 transcripts increased significantly, whereas IL-6⁺ PaSC frequency showed only a non-significant upward trend—consistent with temporal delay of protein expression and/or post-transcriptional regulation, suggesting an initiation rather than fully executed CAF polarization changes. Still, across co-culture formats, KRAS^G12D^ epithelial secretomes reproducibly activated PaSCs and constrained T-cell access.

### TNFα reduces T cell migration and activates pro-tumorigenic fibroblasts

To probe early niche preparation, we integrated our time-resolved KRAS^G12D^ PDLO dataset with the Carpenter et al. single-cell map (‘*Carpenter niche*‘) of donor pancreata spanning healthy tissue and sporadic PanIN-bearing samples, the latter hereafter referred to as ‘*lesion’* [19]. We then used this merged scRNA-seq resource as a virtual pre-cancer atlas to benchmark PDLO states and predict epithelial–stromal/immune communication patterns.

**Fig. 4:**
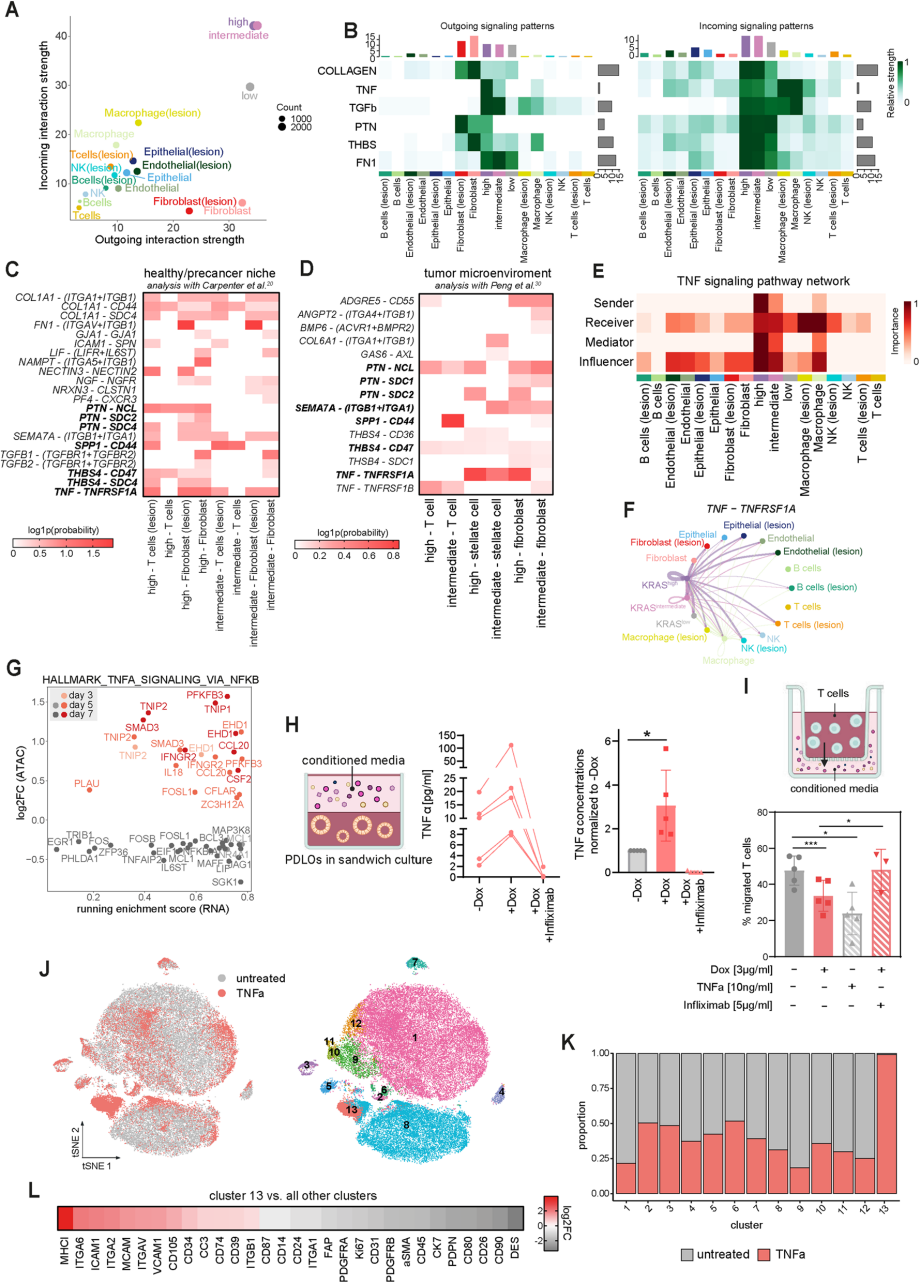
TNFα reduces T cell migration and activates pro-tumorigenic fibroblasts. **A** Scatter plot showing predicted interaction strengths of different cell types derived from pancreatic tissue of healthy donors [19] with and without PanIN lesions and PDLOs. **B** Signaling patterns of selected pathways in different cell types derived from pancreatic tissue of healthy donor [19] with and without PanIN lesions and PDLOs. **C + D** Heatmap showing the interaction probability of ligand-receptor (LR) pairs between PDLOs and cell types derived from pancreatic tissue of healthy donors [19] with and without PanIN lesions (healthy microenvironment, left) and cell types derived from PDAC tissue [33] (tumor microenviroment, right). Only ligand-receptor pairs are shown, which are predicted to originate from either KRAS^high^ or KRAS^intermediate^ but not from KRAS^low^ cells. Overlapping LR-pairs between both analyses are highlighted in bold. **E** TNF signaling patterns in different cell types derived from pancreatic tissue of healthy donors [19] with and without PanIN lesions and PDLOs. **F** Circle plot showing predicted interaction strength of the ligand-receptor pair TNF-TNFRSF1A between different cell types derived from pancreatic tissue of healthy donors[19]with and without PanIN lesions and PDLOs. **G** Core enriched genes from scRNA-seq data set of GSEA against the HALLMARK_TNFA_SIGNALING_VIA_NFKB gene set and corresponding log2FC values from the ATAC-seq data set. **H** TNFα concentrations in PDLO-derived conditioned media from 5 independent pancreatic differentiation experiments (left) and relative levels normalized to the untreated -Dox control (right). Mean ± SD. Paired t-test *, *p* < 0.05. **I** Percentage of migrated T cells towards PDLO conditioned media ± TNFa/infliximab. Mean ± SEM, *n* = 4. One-way ANOVA *, *p* < 0.05; ***, *p* < 0.001. **J** t-distributed stochastic neighbor embedding (tSNE) plot showing murine fibroblasts untreated or treated with TNFα for 72 h before subjected to CyTOF analysis (left) and the respective clusters (right). **J** Proportions of cells per cluster from the t-SNE plot. **K** Log2FC values of markers from cluster 13 compared to all other clusters

Combining the KRAS^G12D^ PDLO atlas with the ‘*Carpenter niche*‘, CellChat analysis revealed a *KRAS*-dose–dependent rise in epithelial connectivity (from low → intermediate/high), particularly for the incoming interaction strength (Fig. 4A). Of note, ‘*lesions’*-comprising samples in the Carpenter dataset were accompanied by broader cellular crosstalk than healthy compartments, with endothelial, epithelial and immune cells presenting an increase in interaction strength (Fig. 4A). While fibroblast connectivity appeared to be not more pronounced in ‘*lesions’*-comprising pancreata than in healthy controls, fibroblasts still had the strongest outgoing signaling strength amongst all niche cells (Fig. 4A). Deconvolution of the strengthened edges revealed potential ECM signaling including COL1A1 → ITGA1/ITGB1, CD44 → SDC4, FN1 → ITGAV/ITGB1, THBS4 → CD47/SDC4, TGFB1/2 → TGFBR1/2 and PTN → NCL/SDC2/SDC4 (Fig. 4B, C). Projecting the PDLO states additionally into an end-stage PDAC atlas [33] (‘*Peng niche*‘) recapitulated the interaction space of the premalignant space to a large extent, with KRAS^intermediate/high^ PDLOs lying closest to malignant ducts regarding interaction strengths (Fig. 4D; Supplementary Fig. 4 A,B).

Albeit we observed also an increase in incoming signal strength upon *KRAS*^*G12D*^ expression, we subsequently prioritized on epithelial ligands as potential outgoing factors that could explain the experimentally observed T-cell shielding effect and PaSC activation, which has been mediated by the secretome of KRAS^G12D^ PDLOs. One of the top ligand candidates that was consistent between both data sets, was *TNF. TNF* outflow rose from KRAS^low^ to KRAS^intermediate/high^ PDLOs and *TNF* reception was high, especially in lesion-associated niche cells (Fig. 4B-E; Supplementary Fig. 4B,C). While interaction of *TNF* with *TNFRSF1A* (also known as *TNFR1*), as the major TNF receptor, was projected for most of the niche cells in the Carpenter et al. single-cell map, the receptor interaction was weaker on T cells in the PDAC space. In the PDAC space, TNFRSF1A appeared to be the more probable receptor on PaSC cells, while signaling to Tcells was predicted via *TNFRSF1B* (also known, as *TNFR2*), a second key TNF receptor (Fig. 4C,D,F; Supplementary Fig. 4D). Moreover, Dox treatment resulted in a significant enrichment of TNFα signaling through the NF-κB pathway (Supplementary Fig. 4E). In addition to the expression profile, chromatin regions of several genes of the TNF signaling pathway were differentially accessible upon *KRAS*^*G12D*^ induction (Fig. 4G).

For compound in vitro testing, we focused, besides TNFα, also on other soluble factors and excluded matrix and transmembrane proteins, such as *ANGPT2* and *SEMA7A*. From the three tested ligands (TNF, SPP1 and PTN) only TNFα showed a promising dose-dependent effect on T-cell migration in a pilot experiment with minimal medium (Supplementary Fig. 4 F). To check if TNFα protein is truly secreted in KRAS^G12D^ PDLOs in a relevant manner, we performed TNFα ELISAs on conditioned media from PDLOs. KRAS^G12D^ induction indeed robustly and significantly increased TNFα secretion (between 1.7 and 5.6-fold), and co-treatment with infliximab, a soluble TNF-neutralizing antibody, attenuated this signal toward baseline, confirming successful TNFα binding blockade at the applied dosage (Fig. 4H). Importantly, parental (KRAS-WT) PDLOs did not elevate TNFα upon Dox exposure (Supplementary Fig. 4G). To investigate the role of TNFα in KRAS^G12D^ PDLO-mediated niche remodeling, we performed functional assays. TNFα addition to healthy PDLO-conditioned media significantly reduced T-cell migration, while the addition of infliximab (TNFα neutralization) to the conditioned medium of KRAS^G12D^-PDLOs was in turn sufficient to reduce T-cell migration (Fig. 4I), supporting the hypothesis that T-cell shielding is mediated by TNFα secretion.

Next, we employed an established and comprehensive cytometry by time of flight (CyTOF)-based CAF profiling panel [38], to analyze the effects of different *KRAS*^*G12D*^-instructed ligand candidates on murine fibroblasts. While other candidates, namely BMP6, PTN, and THBS4, did not affect the composition of fibroblast subpopulations when added to the fibroblast culture medium (Supplementary Fig. 4H), TNFα treatment changed fibroblastic marker expression with a strong increase of cluster 13 (Fig. 4J-L). Compared to all other clusters, this subpopulation expressed increased levels of cell adhesion molecules, such as ICAM1 and VCAM1, as well as CD105 (Fig. 4L), which was previously identified as a marker for tumor-supportive fibroblasts [38]. Moreover, decreased expression of canonical fibroblast markers, such as PDPN,FAP, and the myCAF marker aSMA, indicated an impact of TNFα on fibroblast plasticity. The upregulated markers from cluster 13 could also be found in fibroblasts within a re-processed scRNA-seq data set containing samples from healthy and PDAC donors [19] (Supplementary Fig. 4I,J).

Prompted by the CellChat analysis, where KRAS^intermediate/high^ epithelium emerged as a TNFα source engaging macrophages in tumors (Fig. 4B,E,F; Supplementary Fig. 4B-D), we asked whether TNFα could also be involved in attraction of pro-tumorigenic macrophages at such an early dysplastic stage. For this, we performed a chemotaxis assay on M2-macrophages (Supplementary Fig. 4 K) using conditioned medium of epithelial PDLOs and C5a as positive attractant control. The secretome of KRAS^G12D^ PDLOs (+ Dox) indeed increased M2-macrophage chemotaxis compared to the -Dox control, while CM from parental PDLOs (± Dox) had no effect (Supplementary Fig. 4L). However, this effect did not appear to be TNFα dependent, as chemotaxis was neither increased by recombinant TNFα to -Dox CM nor reduced by infliximab in + Dox CM. In summary, epithelial KRAS^G12D^ activation generated a macrophage-attracting secretome, independent of TNFα.

### Single-cell RNA-seq of PDLO bi-cultures with T cells and pancreatic stellate cells

**Fig. 5:**
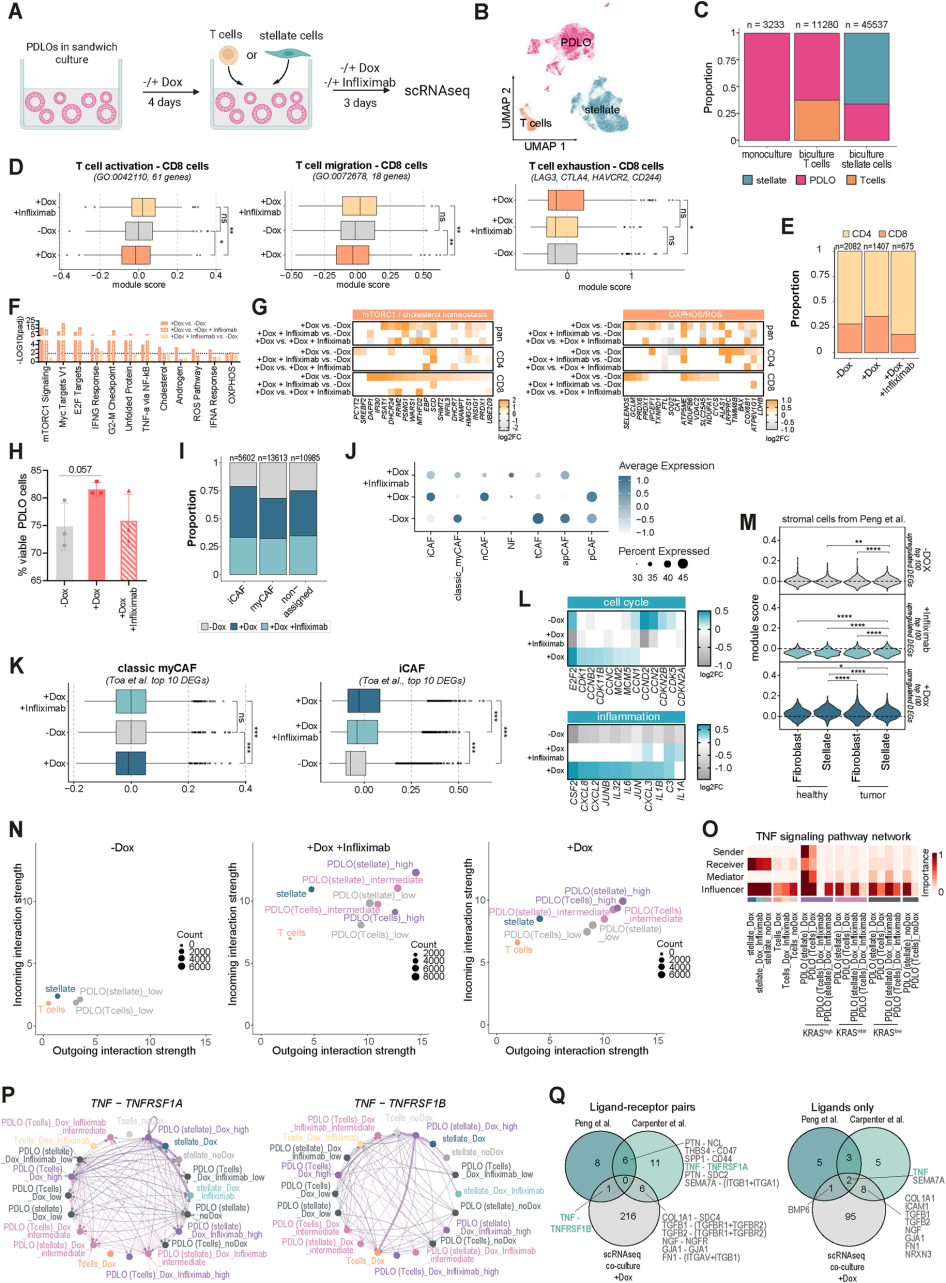
Single-cell RNA-seq of PDLO bi-cultures with T cells and pancreatic stellate cells. **A** Schematic overview of workflow to generate samples for scRNA-seq after co-culture of PDLOs with either T cells or stellate cells. (Scheme was generated using BioRender). **B** UMAP showing PDLOs, T cells and stellate cells from the scRNA-seq analysis. **C** Proportions of PDLOs, T cells and stellate cells within the different culture conditions. **D** Box plot showing the module scores for T cell activation (right), T cell migration (middle) and T cell exhaustion (right) for CD8^+^ T cell subset, visualized as median with corresponding extremes. Wilcoxon test **, *p* < 0.01; ns, *p* > 0.05. **E** Proportions of CD8 and CD4 cells within the different treatment conditions. **F** Significance of HALLMARK gene set enrichment showing the top terms (p.adj < 0.05) using DEGs from the indicated comparisons in all T cells. (IFNG Response = Interferon Gamma Response, Unfolded Protein = Unfolded Protein Response, TNF-α via NF-κB = TNF-α Signaling via NF-κB, Cholesterol = Cholesterol Homeostasis, Androgen = Androgen Response, ROS Pathway = Reactive Oxygen Species Pathway, IFNA = Interferon Alpha Response, OXPHOS = Oxidative Phosphorylation). **G** Heatmap showing log2FC values of DEGs related to mTORC1 Signaling, Cholesterol Homeostasis, ROS and OXPHOS, from indicated comparisons within all T cells (pan), CD8 or CD4 subsets. **H** Viability of PDLOs when co-cultured with T cells assessed by flow cytometry. **I** Proportions of myCAF- and iCAF-like stellate cells annotated by module scores using the gene signatures from Elyada et al. [39]. **J** Dot plot showing module scores of different CAF subpopulations using the top 100 DEGs from Toa et al. [40]. **K** Box plot showing module scores for the stellate cells using the top 10 DEGs for classical myCAFs and iCAFs from Toa et al. [40], visualized as median with corresponding extremes. Wilcoxon test ***, *p* < 0.001; ns, *p* > 0.05. **L** Heatmap showing log2FC values of selectively chosen cell cycle and inflammation-related DEGs within the stellate cells when comparing one condition to the other two conditions. **M** Violin plot showing module score of stromal cells from Peng et al. using the top 100 DEGs of stellate cells co-cultured with PDLOs. **N** Scatter plot showing predicted interaction strengths of the different cell types within the co-cultures. **O** TNF signaling patterns in the different cell types within the co-cultures. **P** Circle plot showing predicted interaction strength of the ligand-receptor pair TNF-TNFRSF1A (left) and TNF-TNFRSF1B (right) between the different cell types within the co-cultures. **Q** Venn diagram showing overlapping ligand-receptor pairs (left) and respective ligands only (right) when comparing the cell–cell communications analysis between PDLOs with pancreatic tissue of healthy donors [19], PDAC tissue [33] and the in vitro co-culture. For this comparison, only ligand-receptor pairs were used, which were predicted to originate from either KRAS^high^ or KRAS^intermediate^ but not from KRAS^low^ cells, and receiving cell populations (expressing the corresponding receptor) were limited to T cells, stellate cells, and fibroblasts

In addition to the in silico predictions of epithelial-niche interaction, we generated a second scRNA-seq dataset from direct bi-cultures employing PDLOs derived from a second KRAS^G12D^ hESC line to demonstrate the robustness of our findings. Using sample multiplexing, we sequenced 10 conditions (*cond.*). built from PDLOs pretreated ± Dox for 4 days to induce KRAS^G12D^, then (for co-cultures) combined for 3 days with primary T cells or PaSCs: PDLO monocultures under − Dox, + Dox, and + Dox + infliximab (*cond.1–3*) and the corresponding co-cultures with either T cells (*cond.4–6*) or PaSCs (*cond.5–9*) under the same treatments (referred as *co-culture data set;* Fig. 5A; Supplementary Fig. 5 A). Next, we analyzed the co-culture dataset of 60,003 cells, comprising 25,590 PDLOs together with 4,208 T cells and 30,205 PaSCs. Joint UMAPs of the resolved three distinct populations with cell type specific marker expression – PDLOs (KRT19), T cells (CD3E), and PaSCs (ACTA2) – confirming proper clustering across conditions (Fig. 5B,C; Supplementary Fig. 5B,C).

To probe a potential doxycycline (Dox) confounder, we additionally included a + Dox monoculture control of parental hESC-derived PDLOs (not carrying a KRAS^G12D^ construct; *cond.10*). We then compared the day-7 monocultures from the time-series (see Fig. 1) with the PDLOs from the co-culture data set. Dox-treated parental PDLOs clustered nearest to untreated (− Dox) KRAS^G12D^ PDLOs in both datasets, and KRAS^G12D^ PDLOs displayed a similar niche-instructive program when contrasted with the parental + Dox control (Supplementary Fig. 5D,E).

*PDLOs in bi-cultures.* PDLOs from the co-cultures clustered according to KRAS^high^ vs. KRAS^intermediate^ vs. KRAS^low^ status from the initial data set (see Fig. 1) underpinning the robustness of the set up (Supplementary Fig. 5 F). To investigate, if PDLO cells in bi-culture show a comparable niche-instructing program as monocultures, we isolated all epithelial KRAS^G12D^ PDLOs (± Dox) of the *co-culture data set* and found a similar distribution of KRAS subpopulations in mono- and bi-cultures (Supplementary Fig. 5G). Relative to KRAS^low^, KRAS^high^ PDLOs exhibited, also in this *co-culture experiment*, a pronounced upregulation of ECM/adhesion and protease components, TGFβ components and selected chemokines, cytokines and its receptors. KRAS^intermediate^ largely mirrored these changes with partly smaller effect sizes, and co-culture primarily altered magnitude rather than direction (Supplementary Fig. 5H).

#### Bi-cultures with T-cells 

In T cell co-cultures, CD8^+^ T cells exposed to + Dox KRAS^G12D^ epithelial PDLOs presented reduced activation and migration signatures alongside higher exhaustion marker expression, findings which are consistent with pro-tumorigenic effects. Notably, TNFα neutralization (through infliximab) counteracted these KRAS^G12D^ effects on functionally related T cell marker expression (Fig. 5D). Intrinsic cell-to-cell variability in the polyclonal PDLO cultures further enabled us to resolve functional consequences that became spatially apparent in the bi-cultures. Here, HA-tag negative (KRAS^G12D^-off) PDLOs were preferentially infiltrated by T cells, whereas adjacent HA-tag positive (KRAS^G12D^-on) organoids were T-cell excluded, demonstrating a KRAS-dependent immune-evasion phenotype that was consistent across multiple donors (Supplementary Fig. 5M). Transcriptionally, KRAS^G12D^ induction slightly increased the proportion of cytotoxic CD8^+^ T cells (Fig. 5E, Supplementary Fig. 5I-L). Furthermore, KRAS^G12D^ induction in PDLOs led to a significant activation of core proliferative pathways (mTORC1, Myc, E2F), functional-related pathways (IFNy, IFNα) and stress-related signatures (ROS, OXPHOS) in T-cells (Fig. 5F, G). Interestingly, nearly all top *hallmark programs* enriched with KRAS^G12D^ mapped to TNFα-responsive biology, (i) highlighting soluble TNFα as a key driver of early T-cell programming and (ii) suggesting tight cross-talk among T-cell instructive signaling hubs. To validate if such transcriptomic changes are reflected on protein level, we performed multiparameter flow cytometry of T cells co-cultured with epithelial PDLOs for 3 days (Supplementary Fig. 5 N). As CD4⁺/CD8⁺ ratios and canonical activation/exhaustion markers did not differ significantly upon KRAS^G12D^ induction or infliximab co-treatment, we concluded that T-cell reprograming is likely nascent and not fully rewired within 3 days of co-culture. Finally, we checked viability of PDLO within bi-cultures and found a trend toward higher viability upon KRAS^G12D^ induction in PDLOs (*p* = 0.057) with a modest reduction with additional infliximab treatment – supporting a potential shielding and anti-inflammatory effect upon KRAS^G12D^ expression (Fig. 5H).

#### Bi-cultures with pancreatic stellate cells

scRNA-seq of PaSCs cultured with epithelial PDLOs resolved 10 Louvain clusters (Supplementary Fig. 6 A,B) that could not be unambiguously mapped to published CAF programs [40] (Supplementary Fig. 6 C), suggesting that PaSC remodelling into a CAF environment has not been fully completed. To chart transcriptional changes and nascent CAF programs, we calculated module scores (average expression levels of gene sets subtracted by the averaged expression of a randomly chosen control gene set) for each cell. Cells were then assigned to the gene signature for which they had the highest module score in case the score was additionally higher than the mean of all cells. Probing classical myCAF/iCAF signatures from Elyada et al. [39] revealed an increase into iCAF states upon KRAS^G12D^ induction, which appeared partially rebalanced by infliximab (Fig. 5I).

Using the more granular CAF signature set from Toa et al. [40], + Dox increased the fractions of iCAF, nCAF, and pCAF states at expense of apCAF and myCAF states (Supplementary Fig. 6D). nCAFs represent a normal-like transitional state bridging fibroblasts and CAFs, whereas pCAFs are proliferative [40]. Notably, TNFα blockade with Infliximab directionally (partially) reversed the + Dox-induced shifts in iCAF, nCAF, myCAF, and apCAF, while the pCAF increase was not mitigated. Enrichment was observed in module score-based cell annotation (Supplementary 6D), but also overall expression level of the program (Fig. 5J) and infliximab was again able to partly shift cellular states toward the -Dox distribution (Fig. 5I,J; Supplementary Fig. 6D). Notably, the shift from classical myCAF to iCAF states was still evident when using only the top 10 DEGs described by Toa et al. [40] (Fig. 5K), emphasizing the robustness of the changes.

Additional DE gene analysis within our PaSCs revealed upregulation of inflammatory mediators (e.g., *CSF2, CXCL8/3/2, IL6/1B/JUN*) and cell-cycle genes (*E2F2, MCM2/5, CCN1/2/D2*) for the + Dox condition, which was attenuated by TNF blockade (Fig. 5L). Moreover, expression levels of the top 100 DEGs from the + Dox condition were also upregulated within the stromal compartment of the data set from Peng et al. [33] (healthy vs. PDAC tissue), while this is not observed for the -Dox or + Dox + infliximab condition (Fig. 5M), underpinning the observed relevance of transcriptional changes.

#### Cell–cell communication in bi-cultures

CellChat analysis showed a KRAS^G12D^-dependent surge in intercellular signaling not rescued by TNFα blockade, with + Dox cultures exhibiting stronger outgoing/incoming interactions across PDLOs, PaSCs, and T cells than –Dox. Notably, even KRAS^low^ cells exceeded –Dox PDLOs, indicating a globally altered ligand–receptor landscape in the respective sample/condition ecosystem (Fig. 5N). Examining pathway differences in ligand–receptor communication highlighted, besides TNFα, several candidates of interest: immunomodulation (MIF), chemokines (CXCL), ECM/remodeling (COLLAGEN, FN1, TGFB), growth factors (PDGF, EGF), and angiogenic cues (VEGF). While these nominate additional mechanisms by which oncogenic KRAS sculpts the niche, the present study focused on the TNFα axis for functional dissection (Supplementary Fig. 6E). While KRAS^High^ PDLOs were again dominant TNFα senders, with infliximab treatment successfully diminishing this signal, signals from KRAS^Intermediate^ PDLOs were less evident than in our first data set (Fig. 5O,P; compare Fig. 4E, Supplementary Fig. 4 C). This might be explained by lower penetrance of KRAS^G12D^ induction in the co-culture data set, and a therefore more diluted KRAS^Intermediate^ PDLO population. Of note PaSCs were recognized as major receiver of TNFα-signaling, while incoming signals were weaker for T cells (for both TNFRSF1A/B receptors) compared to the in silico predicted interactions (Fig. 5O, also compare to Fig. 4E, Supplementary Fig. 4 C).

As a final convergence step, we rigorously compared LR pairs from the predicted cancer niches (‘Carpenter niche‘ and ‘*Peng niche*‘) with our co-cultures. This stringent intersection yielded a small, specific overlap; at the ligand level only TNF (and SEMA7A) recurred across all datasets. Aligned with our functional perturbations, this convergence supports a model in which epithelial KRAS leverages TNF to prime PaSCs toward iCAF-like inflammation and modulate early T-cell programs. It also indicates that the KRAS^G12D^ secretome harbors further niche-programming factors that merit functional follow-up (Fig. 5Q).

### Translational validation across various models

**Fig. 6:**
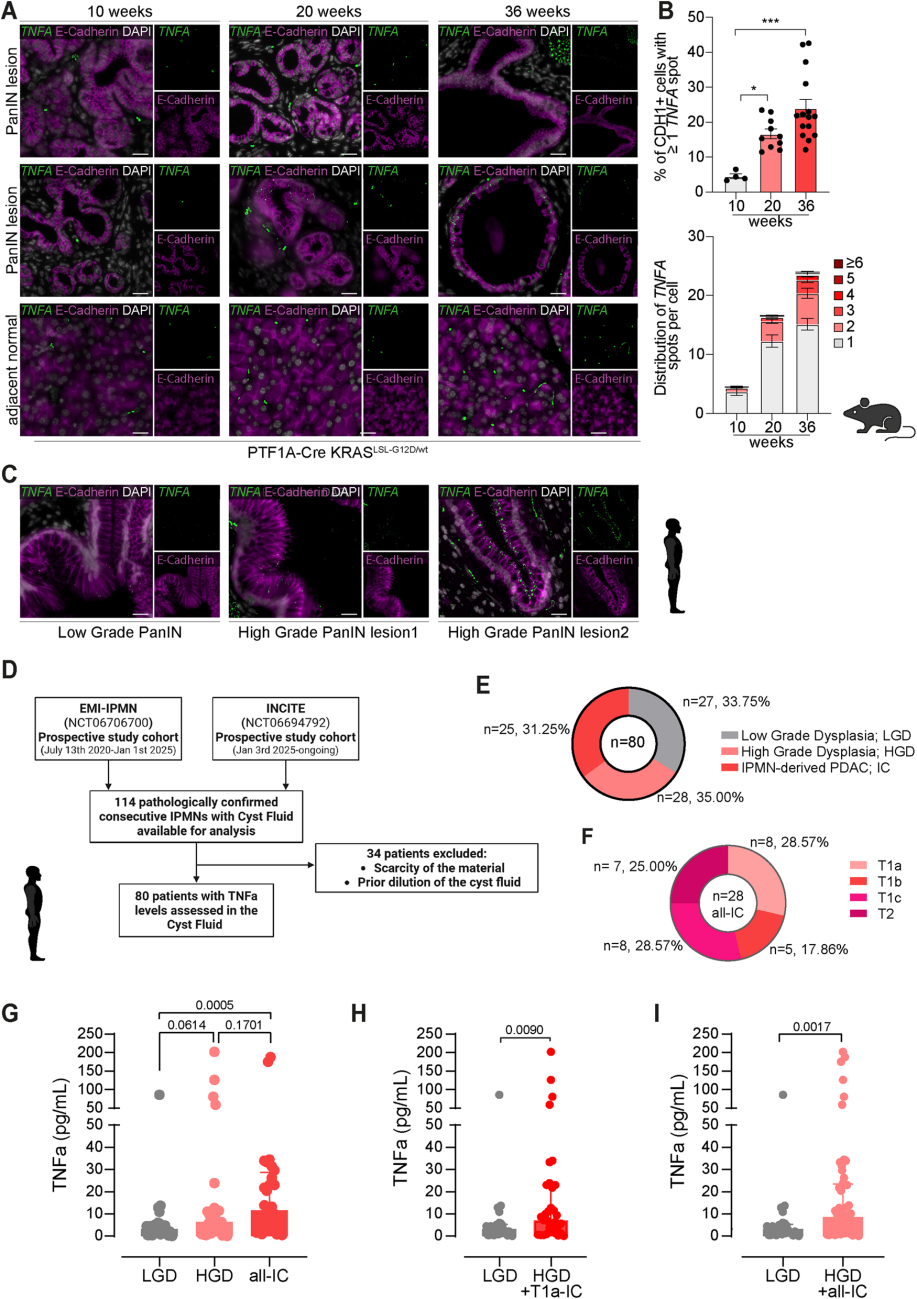
**A** Representative RNAscope images combined with E-cadherin immunofluorescence showing Tnf-α mRNA puncta in epithelial cells of PanIN lesions from *Ptf1a-Cre;Kras*^*LSL−G12D*^*/wt* (KC) mice aged 10, 20, and 30 weeks. Subcellular Tnf-α spots indicate discrete transcript signals, while erythrocytes display non-specific pan-staining artifacts. Subcellular Tnf-α expression was, in total, quantified in 4553, 16,740, and 30,084 epithelial cells for the respective 10, 20, and 30 week time points. **B** Respective quantification of (**A**). The upper bar graph depicts the percentage of epithelial cells harboring ≥ 1 Tnf-α spot, the lower bar graph shows the detailed distribution of Tnf-α spot counts per cell (in %). Statistical analysis was performed using one-way ANOVA followed by Tukey’s multiple-comparison test; n = number of PanIN regions analyzed across different mice; **C** RNAscope–immunofluorescence images of human PanIN-containing pancreatic tissues illustrating TNFA expression within epithelial compartments; **D** Pathological diagnosis of the 80 patients with IPMN and IPMN-derived invasive PDAC included in the final analysis; **E** Lesion and tumor distribution of the investigated 80 patients. **F** Tumor stage distribution of the 28 patients with IPMN-derived invasive PDAC; **G** Levels of TNF-α in cyst fluid stratified according to the degree of dysplasia; **H** Comparison of cyst fluid TNF-α levels between patients with low-grade dysplasia and those with high-grade dysplasia or minimally invasive cancer (T1a only); **I** Comparison of cyst fluid TNF-α levels between patients with low-grade dysplasia and those with high-grade dysplasia or invasive carcinoma arising from IPMN. Statistic test: one tailed Mann Whitney

PDAC develops through well-defined precursors—PanINs and cystic neoplasms—that progress from low- to high-grade dysplasia (LGD/HGD), with ductal mutations such as KRAS^G12D^ initiating cystic programs (either intraductal papillary mucinous neoplasm (IPMN) or mucinous cystic neoplasm (MCN)) amenable to early interception [14, 21, 41]. To provide translational in vivo validation for our preclinical findings, we first analyzed archived FFPE tissue from the pancreas-specific KRAS^G12D^ mouse model (KC), sampled at 10, 20, and 36 weeks spanning progressive low- to high-grade PanINs [42]. Using RNAscope to detect Tnfα (TNFα) transcripts, we quantified subcellular TNFα spots per E-Cadherin (CDH1) positive cell, and observed clear epithelial TNFα signals significantly increasing with dysplasia over time (Fig. 6A,B). To corroborate these findings in humans, we applied the same approach to PanIN-comprising human pancreata and again detected clear epithelial TNFα signals (Fig. 6C).

Finally, we analyzed a prospective cohort of IPMN patients (*n* = 80 total with available TNFα data from the EMI-IPMN and INCITE trials; Fig. 6D–F), quantifying TNFα in undiluted intraoperatively collected cyst fluid. The cohort was pathologically confirmed and structured to represent the progression continuum (Fig. 6E): 33.75% (*n* = 27) were Low-Grade Dysplasia (LGD), 31.25% (*n* = 25) were High-Grade Dysplasia (HGD), and 35.00% (*n* = 28) were IPMN-derived Invasive Cancer (IC). This IC subgroup was further classified by tumor (T) stage (Fig. 6F), including 28.57% (*n* = 8) with T1a (minimal/micro-invasion). Cyst fluid TNFα levels exhibited a stepwise escalation corresponding to increasing disease severity (Fig. 6G). TNFα was lowest in LGD (median 3.21pg/mL, *n* = 27) and rose in HGD (median 6.35pg/mL, *n* = 25; *p* = 0.0307 vs LGD). Levels peaked in IPMN-derived Invasive Cancer (IC; median 11.65pg/mL, *n* = 28), demonstrating a highly significant increase when compared to LGD (*p* = 0.0003). Furthermore, analysis of stage transitions confirmed significance for relevant comparisons (Fig. 6H,I). TNFα was significantly higher in HGD plus microinvasive carcinoma (T1a) versus LGD (*p* = 0.0090) and increased further when overt invasive carcinomas were combined with HGD (HGD + IC; *p* = 0.0017 vs LGD). These data provide robust clinical evidence that cyst-fluid TNFα levels consistently peak with invasion, supporting the clinical relevance of an epithelial TNF program that intensifies along the IPMN–PDAC continuum. Considering that the cyst fluid is compartmentalized and isolated by the epithelial cells, it strongly underpins the transformed epithelium as the likely source of TNFα secretion.

## Discussion

Our study demonstrates how an oncogenic signal such as KRAS^G12D^ in the human ductal lineage rapidly reprograms epithelial states and initiates a niche-preparatory communication program. Using hESC-derived pancreatic duct-like organoids (PDLOs) profiled by single-cell transcriptomics and chromatin accessibility, we captured time-resolved epithelial remodeling at the onset of preneoplastic transformation—processes not accessible in conventional models—and mapped these states onto healthy, lesion, and cancer in-silico niches. Across multiple layers, we consistently identified TNF–NF-κB, IL-6/JAK–STAT3, TGF-β, and ECM/integrin signaling axes as potential drivers of early niche remodeling. Complementary single-cell co-culture experiments involving either T cells or pancreatic stellate cells (PaSCs) converged on a ligand/receptor-driven program that links inflammatory signals to extracellular matrix remodeling. These likely interconnected processes, typically regarded as hallmarks of established PDAC [43], are revealed here as initiating events during tumor development.

Distinct, yet pathobiologically convergent, epithelial states arose rapidly in PDLOs, likely reflecting graded KRAS^G12D^ dosage from non-uniform piggyBac insertions; despite days of induction, they bridged healthy–malignant ductal programs, aligned with survival and TME composition, and recapitulated advanced ligand–receptor patterns. Previous in vivo studies have shown that inflammatory cues can “license” KRAS-mutant pancreatic cell subpopulations to activate oncogenic programs before malignant transformation [15, 16]. Consistent with this, a recent preprint integrating lineage tracing with single-cell and spatial transcriptomics mapped tumor initiation and identified a discrete KRAS^G12D^ progenitor-like epithelial population that concurrently engaged senescence/SASP-like programs and oncogenic signaling to remodel an immune-shielded niche—an effect reversed by KRAS inhibition but amplified and reprogrammed upon p53 loss [32]. Together, these findings support a unifying model in which early, plastic ductal states preconfigure late-stage TME architecture through shared and therapeutically tractable signaling nodes, including co-upregulated TGF-β/TNFα pathways, ECM–receptor signaling, and cytokine-mediated epithelial–stromal crosstalk.

Functionally, we prioritized the secretory arm of an epithelial-centered communication hub at tumor initiation: in PaSC interactions, KRAS^G12D^ epithelium rapidly enhanced fibroblast growth and activation, with PaSCs upregulating CAF markers such as *IL6* or *CXCL2* and expanding nCAF, pCAF, and iCAF states at expense of myCAF and apCAFs marker expression [40]. KRAS-secretome exposure consistently induced iCAFs, but myCAF readouts were mixed: conditioned media upregulated canonical myCAF markers, whereas CyTOF and bi-cultures suggested myCAF repression. We infer that this reflects nascent, modality-dependent programming. Longer culture and deeper downstream analyses will be needed to define the stable myCAF/iCAF equilibrium in future studies.

TNFα inhibition by infliximab was capable of partially reversing KRAS^G12D^-triggered changes aligning with previous work that TNFα inhibition attenuates desmoplasia/inflammation and improves therapy response [44]. Mechanistically, a KRAS → NF-κB → TNFα axis emerges as a signaling checkpoint that translates epithelial oncogenic dosage into stromal activation, including induction of fibroblast adhesion molecules (ICAM1, VCAM1) that can modulate lymphocyte interactions and trafficking dynamics within the niche [45–48]. Consistent with this model, dampening canonical NF-κB constrains fibrosis, inflammation, and PanIN formation in KRAS^G12D^ mice, while anti-TNF strategies reduce desmoplasia and inflammatory tone and enhance therapeutic responses [44]. Importantly, we do not claim that TNFα is the sole mediator of KRAS^G12D^-driven early niche remodeling; rather, we resolve this mechanistically and, for the first time, demonstrate it in a human, non-rodent early-dysplasia–mimicking model. We acknowledge that additional epithelial and stromal cues—candidates nominated in our dataset—likely cooperate with or counterbalance TNFα across multiple axes of CAF specification and early immune interference.

Within the immune compartment, we focused on the impact of an oncogenic epithelium on T-cells, and found a shielding effect orchestrated by paracrine signaling of KRAS^G12D^ PDLOs. This effect was robust across conditioned medium, chip-based T-cell flow, and transwell experiments and appeared to be mainly TNF dependent. Consistent with immune evasion, live–dead assays in direct T-cell co-cultures showed a modest survival/proliferation advantage for KRAS^G12D^ PDLOs. Neither exposure to the epithelial KRAS^G12D^ secretome nor 4-day co-culture shifted T-cell subset frequencies (CD4/CD8; activation/exhaustion). However, transcriptomics revealed increased mitochondrial and proliferative programs with altered functional markers—evidence of nascent, incomplete remodeling – while overall T-cell functionality was impaired. Together, this constitutes an incipient immune-evasion signature that echoes advanced PDAC and supports a rapidly evolving contest between immunity and precursor lesions over clearance versus malignant transformation. In particular, we want to highlight that T-cell exclusion effects outweighed potential attraction cues in our epithelial KRAS^G12D^ PDLO T-cell experiments, suggesting that T-cell attraction to precancer lesions, that occurs in vivo in a complex ecosystem, is presumable triggered by other cellular players such as stromal and myeloid cells. Despite not directly investigated in this study, our data has potential implications to understand immune cell isolation and desertation in a stroma-rich environment in PDAC [49, 50].

Beyond stromal and T-cell priming, KRAS^G12D^-PDLO conditioned medium increased chemotaxis of pro-tumor M2-polarized macrophages, apparently TNFα-independent—pointing to parallel, interconnected recruitment routes. This suggests that TNFα blockade alone will be insufficient and argues for combinations targeting chemokine networks and mechano–TGFβ circuits. The role of TNFα in PDAC is stage- and context-dependent: TNFA is enriched in anti-tumor macrophages and suppressed by IL-13/IL-4 in M2 cells [51, 52]. Consistently, macrophage-derived TNFα induced ferroptosis and showed partial anti-tumor effects in immunodeficient xenografts [37], whereas in immunocompetent settings exogenous TNFα increased tumor growth [53, 54]. In line with our data, these discrepancies indicate that the pro-tumorigenic effect of TNFα engages niche interaction and immune evasion despite potential direct tumoricidal effects in reductionist models. Mechanistically, macrophage TNFα can drive tumor de-differentiation/aggressiveness [55], reduce CD8^+^ infiltration and promote anti-inflammatory tone [56, 57], and upregulate PD-L1 on cancer cells [58], but also increase epithelial invasiveness [54]. We did not dissect epithelial-intrinsic TNFα effects, yet our in silico analysis predicted KRAS^G12D^ PDLOs as both TNF senders and receivers, suggesting an autocrine component. Functionally, TNFα inhibition did not improve PDLO survival, arguing against an autocrine cell-death route. The partially contrasting effects of TNFα likely reflect a critical spatial dimension of signaling, where the cytokine's function is dictated not merely by its concentration but fundamentally by its cellular source (epithelial vs. immune cells) and subsequent local receptor engagement [59, 60]. Supporting the concept that the oncogenic epithelium acts as an early niche-programming factor, our translational data show that cyst-fluid TNFα levels rise stepwise from low-grade to high-grade dysplasia and invasive cystic cancer, echoing TNFα expression trends in progressing murine and human PanIN lesions. Together, this strongly implicates epithelial-derived TNFα in early malignant transformation and suggests that the overall pathological outcome is determined by coexisting autocrine and paracrine TNFα signaling across epithelium and immune cells, with final effects contingent on dose, timing, and auxiliary pathways.

From a translational perspective, three insights emerge. First, *timing*: the rapidity with which KRAS^G12D^ established ECM–inflammatory communication suggests that interception windows exist before stable desmoplasia and entrenched immune evasion are fully established. Second, *targeting logic*: our perturbations studies nominate TNFα as an actionable hub linking epithelial transformation to CAF priming and T-cell trafficking restraint, with putative combination strategies guided by additional niche-instructive programs. Third, *biomarkers*: cyst-fluid TNFα scaled with IPMN dysplasia, supporting the feasibility of epithelial-proximate, fluid-based readouts that mirror early niche programming and could guide interception or surveillance strategies. While beyond the scope of the present study, integrating TNFα-centric metrics with ductal state classifiers and secretome signatures could enable stratification of precursor lesions on imminent niche activation rather than morphology alone.

### Strengths and scope

A key strength of this study is its multi-layered integration: single-cell RNAseq and ATACseq, defined epithelial–stromal/immune co-cultures, and quantitative secretome assays, all anchored by functional perturbations (e.g., infliximab, recombinant TNFα) that rigorously establish necessity and sufficiency for critical signaling axes. The use of a human ductal lineage provides direct relevance to PDAC evolution and complements findings from rodent-based models. Importantly, we connect in vitro dynamics to human precursor lesions via cyst-fluid TNFα, providing a direct clinical correlate to the epithelial-niche hypothesis and reinforcing the translational significance of our findings.

### Limitations

Our piggyBac/tetracycline system overexpresses KRAS^G12D^ and does not fully recapitulate endogenous, stepwise dosage ramping. Although pERK amplitudes and downstream programs fall within ranges reported for PDAC models, the high copy-number and artificial gene locus context remains a caveat. We focused specially on KRAS^G12D^—the most prevalent PDAC allele—without incorporating common co-drivers or tumor-suppressor losses (TP53, CDKN2A, SMAD4), which likely modulate early epithelial plasticity and niche programming [50, 61]. Temporally, our analyses captured rapid initiation events but did not encompass the denser, longer courses needed to fully chart state transitions, feedback control, and reversibility under perturbations. Ecosystem complexity is also limited: while controlled bi-cultures (epithelium–PaSC; epithelium–T cell) provide mechanistic insight, they do not capture higher-order interactions. Future tri-/quadri-culture-systems incorporating myeloid and endothelial compartments, organotypic systems, and in vivo validation will be essential to refine the causal hierarchy and support combinatorial intervention strategies. For myeloid biology, input macrophages were M2-skewed and assays emphasized chemotaxis rather than durable repolarization; therefore, conclusions about macrophage plasticity remain provisional. Finally, although matched Dox-only controls across functional and single-cell assays showed no KRAS-like phenotypes, tetracycline systems can exert subtle, context-specific effects, that merit confirmation in Dox-free, locus-knock-in frameworks.

## Conclusions

Within days of in vitro KRAS^G12D^ induction, plastic human ductal epithelium begins to architect the pancreatic niche. Concordant ATAC/RNA/secretome shifts delineate an ECM–inflammatory module centered on TNFα, coupling epithelial KRAS dosage to PaSC activation and T-cell shielding. Rising cyst-fluid TNFα across IPMN grades underscores clinical relevance and nominates TNFα-centric axes for early interception (± checkpoint co-targeting; stromal/ECM modulators). Our co-culture single-cell platform enables testing of co-mutations, temporal control points and reversibility, higher-order ecosystem models, and generalization across precursor lesions—toward precision interception rooted in epithelial state and early niche programming to prevent PDAC.

## Methods

### Cultivation of hESCs and PaSCs

The human embryonic stem cell (hESC) line HUES8 (Harvard University; RRID: CVCL_B207) was used in this study, with culture and differentiation procedures into the pancreatic lineage approved by the Robert Koch Institute (79th Authorization under the Stem Cell Act, AZ 3.04.02/0084). Cells were maintained on hESC-qualified Matrigel-coated plates (Corning) in mTeSR Plus medium (STEMCELL Technologies) at 5% CO₂, 5% O₂, and 37°C. Media was changed every other day, and cells were passaged twice a week at a 1:8 split ratio, as described previously [24, 25, 29].

Human pancreatic stellate cells (PaSCs) derived from a chronic pancreatitis patient and immortalized by the catalytic subunit of hTERT and SV40 large T antigen were kindly provided by Prof. Matthias Löhr (Karolinska Institute). Cells were cultured in DMEM (Gibco) supplemented with 10% FBS (PanBioTech) and P/S (100 IU/mL penicillin and 100 µg/mL streptomycin sulfate) at 37 °C under a 5% CO_2_ atmosphere.

### Genetically engineering of cells

The Dox-inducible KRAS^G12D^ overexpression line was generated previously using a piggyBac transposon system [25]. In brief, a multi-step PCR approach and gateway cloning were performed to generate PB-TAC-ERP2-(N-HA)KRAS_G12D plasmid, which was introduced to HUES8 cells by nucleofection (Lonza). Cells were co-transfected with a transposase-expression vector (SBI Biosciences #PB200PA-1) to enable integration into the genomic DNA, and successfully targeted clones were enriched by puromycin selection.

To label the pancreatic stellate cells fluorescently, we introduced a pLac eGFP vector via lentiviral transduction. Therefore, lentiviral particles were produced in LentiX-cells. The cells were seeded in 10-cm dishes. Once they reached a confluency of 85–95% they were transfected. For this, 415 µl of DMEM were mixed with 8 µg of pLac eGFP vector, 5.5 µg psPAX2 vector and 2 µg pMD2 vector and 70 µL of 1 mg/mL PEI in 20 mM HEPES and 150 mM NaCl at pH 7.4 and incubated at room temperature for 10 min. During the incubation 6.5 mL of DMEM were added to the dishes. 1 mL of DMEM were added to the DNA/PEI mix and then added to the dish. After incubating for 4 h the complete medium was replaced. On day 2 and 4 the medium was changed and the old medium stored at 4°C. The pooled virus-containing medium was centrifuged at 1000 × g for 2 min to pelletize remaining cells, the supernatant then sterile-filtered with a 0.45-µm filter. To concentrate the virus one part of Lenti-X concentrator were added to three parts of virus supernatant. This mix was incubated at 4 °C for 1 h and then centrifuged at 1500 × g for 45 min at 4°C. The supernatant was discarded and the pellet was resuspended in 1 mL DMEM. The PaSCs were then transduced with 50 µL of virus concentrate and after 2 days Neomycin was added to select transduced cells for 7 days.

### Pancreatic differentiation of stem cells

Pancreatic differentiation of hESCs was achieved as previously described [24, 29]. Therefore, hESCs were seeded on Matrigel (Corning) or Ultimatrix (R&D Systems) coated plates, and the differentiation process was initiated after 24 h, at 70—95% confluency, by adding day 0 media. The medium with the corresponding stage-specific compound composition [29] was changed daily. At pancreatic progenitor stage (PP, day 13), cells were allocated for magnetic-activated cell sorting (MACS) to enrich for a GP2-positve population and thereby improve PP purity. PPs were then reseeded in growth factor reduced (GFR) Matrigel or Ultimatrix to allow formation of pancreatic duct-like organoids (PDLOs), either in sandwich cultures, as previously described [24], or in 50-µL Matrigel or Ultimatrix domes with 40,000 cells/dome. Media change was performed every 3 to 4 days. If not stated otherwise, experiments including co-cultures were performed using PDLO media, comprising BE3 (MCDB131, 1% Glutamax, 3.3 g/L glucose, 1.754 g/L sodium bicarbonate, 20 g/L fatty acid free BSA, and 0.5% insulin-transferrin-selenium-X, 1% P/S) supplemented with 50 ng/mL FGF10, 50 ng/mL EGF, 10 mM nicotinamide, and 10 μM ZnSO_4_. PDLOs were passaged once after 27 to 29 days of differentiation [24], allowing reformation of organoid structures for 2 to 4 days before inducing *KRAS*^*G12D*^ expression by adding 3 µg/mL Dox (Sigma). For re-seeding or harvesting of PDLOs, Matrigel or Ultimatrix was dissolved with 1 mg/mL collagenase/dispase (Roche) solution for 2 h at 37 °C, before organoids were dissociated into single cells in Accutase solution (Merck) for 30 min at 37°C.

### Preparation and sequencing of PDLOs and co-cultures for scRNA-seq analysis

For the time-series data set, samples were harvested 1, 3, 5, and 7 days after the start of Dox treatment. Initially, untreated PDLOs from day 1 and day 7 were included as controls. However, the day 1 control sample was later excluded from the analysis due to poor sample quality. Single cells were then cryo-preserved in DMEM (Gibco) with 10% heat-inactivated FBS and 10% DMSO. For sequencing, cells were thawed and dead cells were removed using the Dead Cell Removal Kit (Miltenyi Biotec). An RNA library was prepared using the 10 × Genomics Chromium Single Cell 3’ Library and Gel Bead Kit v3.1, and the amplified cDNA library was sequenced on an Illumina NovaSeq 6000 S2 flow cell. To annotate the inserted KRAS^G12D^ construct, the corresponding sequence was added to the GRCh_38 genome. The raw sequencing files were used as input for the CellRanger pipeline (10 × Genomics), which allowed demultiplexing, alignment, barcode and UMI counting, as well as filtering and correction.

For the co-culture data set, PDLOs were treated with doxycycline for 4 days, before adding T cells or stellate cells. In one condition, 10 µg/ml infliximab was added to the dox-treated cultures to neutralize soluble TNFα. After 3 days of co-culture and infliximab treatment, samples were harvested. Cells were fixed using the GEM-X Flex Sample Preparation v2 Kit (10 × Genomics) according to the manufacturer's protocol (CG000782). Multiplexed libraries were prepared following the 10 × Genomics user guide “GEM-X Flex Gene Expression for Multiplexed Samples” (CG000787) using the Chromium X (10 × Genomics) and the Chromium Human Transcriptome Probe Set v1.1.0 (10 × Genomics). A self-designed customized probe was added, targeting the sequence of the mCherry reporter of the KRAS^G12D^ construct (LHS-CCTTGGCACCCGAGAATTCCAATTACGGGGCCGTCGGAGGGGAAGT and RHS-phospho-TGGTGCCGCGCAGCTTCACCTTGTA-ACGCGGTTAGCACGTA-NN-XXXXXXXX-CGGTCCTAGCAA). Two independent sequencing runs were conducted. The first run included all samples of mono- and bi-cultures, while bi-cultures were additionally sequenced in a second run. Sequencing was performed on a NovaSeq 6000 instrument (Illumina). Flow cell demultiplexing was achieved by using the BCLConvert tool (v4.2.4, Illumina), followed by further processing with the CellRanger Multi -pipeline (v9.0.0, 10 × Genomics).

### Preprocessing of scRNA-seq data

For the time-series data set, using the Python package Scanpy v1.7.0 [62], each sample's raw count matrix was filtered to include only cells with less than 15% mitochondrial counts and more than 1200 genes overall, excluding genes detected in less than 3 cells. The different AnnData objects were then concatenated, normalized and log transformed. Further downstream analysis was mainly performed in R, for which the h5ad file was converted to h5seurat format using SeuratDisk v0.0.0.9020.

Within the co-culture scRNA-seq data set, discrepancies were observed between the raw counts of individual probes targeting the same gene (e.g. mean raw count KRT19: probe 1 = 3.4007, probe 2 = 0.0225, probe 3 = 0.0059). To minimize potential bias due to variable probe performance, the raw probe matrix was filtered to exclude probes with mean raw counts (across all cells) below 20% of the mean raw counts of the best-performing probe for that gene. From initially 54,582 probes, 37,651 (first sequencing run) and 39,069 (second sequencing run) remained, which averages to approximately 2 probes per gene. The raw counts of the remaining probes were then aggregated by computing the mean count per gene and cell. Using Seurat v5.0.0, the two sequencing runs were merged and cells with less than 300 or more than 8000 unique features as well as more than 30% mitochondrial counts were excluded.

### KRAS^G12D^-dependent grouping of cells and Keras/TensorFlow multilayer deep learning

Cells of all time points within the time-series scRNA-seq data set were grouped based on their *KRAS*^*G12D*^ expression. Therefore, the *KRAS*^*G12D*^ expression values were extracted from the normalized count matrix of the Seurat object and the R package stats v4.2.2 was used to perform distance matrix computation (*dist*), followed by hierarchical clustering (*hclust*) and k-means grouping (*cutree*, k = 3). We employed Keras/TensorFlow deep learning model to identify classifiers and predictors in the single-cell sequencing data set. Details on model setup have been previously described [63]. Briefly, we used an initial layer of 16 units, three hidden layers (16 units) and a final output layer of 3 units (multiclass output). A glorot uniform initializer and hyperbolic tangent activation were used for kernel initialization and activation, respectively. We employed softmax in the final prediction layer. A stochastic gradient descent optimizer was used for model optimization along with a categorical cross-entropy loss measurement. Model was trained with 200 iterations using 65% of the dataset (the other 35% was used for validation).

To annotate KRAS^high^, KRAS^intermediate^, and KRAS^low^ cells within the co-culture scRNA-seq data set,the log-normalized mCherry expression values from PDLO cells were extracted, and k-means grouping (k = 3) was performed as described above for the time-series data set. The mCherry gene probe was detected in only 20% of the Dox-treated cells, despite expected fluorescence signals (Suppl. Figure 5 A). To exclude bias based on gene-probe performance, the cells lacking detectable mCherry signal were additionally annotated by label transfer from the time-series data set using Seurat’s anchor-based transfer procedure. After label transfer, 45% of cells in the Dox-treated conditions were annotated as KRAS^high^ or KRAS^intermediate^.

### Scaling, dimension reduction and differential gene expression analysis of scRNA-seq data

Further processing of the time-series and co-culture scRNA-seq data sets were performed using the R package Seurat [64–66] v 4.2.0–5.1.0 and the packages ggplot2 v3.4.0–3.5.1 [67] and SCpubr v2.0.2 [68] were additionally implemented for visualization. For the time-series data set, the deep learning classifiers (1452 genes) were used as *VariableFeatures*, on which data was scaled. Principal component analysis (PCA) was computed on the first 50 PCs and nearest-neighbor graph construction as well as Uniform Manifold Approximation and Projection (UMAP) dimensional reduction were done using 30 dims. To determine differentially expressed genes (DEGs) between groups, the *FindMarkers* function was called, using DESeq2 and setting a threshold of 0.2 for log2FC and < 0.05 for the p-value.

The raw counts of the co-culture scRNA-seq were log-normalized (*NormalizeData,* default) and scaling was performed based on the top 2000 variable genes. PCA, nearest-neighbor graph construction as well as Uniform Manifold Approximation and Projection (UMAP) was computed using the first 30 PCs. The individual cell types (PDLOs, T cells and stellate cells) were annotated based on the cluster distribution throughout the different culture conditions, e.g. clusters which were only present in the monoculture were assigned as PDLOs, additional clusters in the bi-culture conditions were then designated as T cells or stellate cells. The cell-type annotation was then further validated by cell-type-specific marker expression (e.g., KRT19 for PDLOs, CD3E for T cells, and ACTA2 for stellate cells). The individual cell types were then subsetted and investigated separately**:**

#### PDLOs

For the PDLO subset, cells were again log-normalized (*NormalizeData*, default) and scaled using the deep learning classifiers trained on the time-series data set (1452 genes). PCA was computed on the first 50 PCs, and nearest-neighbor graph construction, as well as UMAP dimensional reduction, were done using the first 10 dimensions. To determine DEGs, raw counts were normalized and scaled using sctransform v0.4.1 [69, 70], followed by calling the *FindMarker* function with a threshold of 0.2 for log2FC and < 0.05 for the p-value. To compare the PDLOs from the co-culture data set to the PDLOs from the time-series data set, both data sets were merged, followed by anchor-based CCA integration (*IntegrateLayers*). Nearest-neighbor graph construction and UMAP dimensional reduction were then again conducted on the integrated data set, using the first 30 dimensions. To visualize the similarity between the treatment conditions as well as the KRAS groups between both data sets, the Seurat function *BuildClusterTree(reduction* = *"integrated.cca")* was utilized.

#### T cells

The T cell subset was log-normalized and scaled (*NormalizeData* and *ScaleData*, default). PCA was computed on the first 50 PCs, and nearest-neighbor graph construction, as well as UMAP dimensional reduction, were done using the first 10 dimensions. Cluster 5 (44 cells) did not express relevant levels of CD3 and was therefore excluded from further downstream analysis. The other clusters were then assigned to CD4 or CD8 T cell subsets based on the respective marker expression. Using sctransform v0.4.1 [69, 70], raw counts of all T cells, of the CD4 and of the CD8 subsets were again normalized and scaled, before calling the *FindMarker* function to determine DEGs.

#### Stellate cells

The stellate cells were log-normalized and scaled (*NormalizeData* and *ScaleData*, default). PCA was computed on the first 50 PCs, and nearest-neighbor graph construction, as well as UMAP dimensional reduction, were done using the first 30 dimensions. DEGs were identified with *FindMarkers* on the sctransformed counts.

### Gene set enrichment, KEGG pathway and gene ontology analysis

Gene set enrichment analysis (GSEA) was conducted using the GSEA desktop software v4.3.3 [71, 72], explicitly utilizing the GSEAPreranked tool. Therefore, differentially expressed genes were ranked according to the negative base-10 logarithm of their p-values, multiplied by the sign of the corresponding log2-foldchange. For KEGG pathway and Gene Ontology analysis, the web-based interface Enrichr [73] was used. The Gene-Concept Networks (cnet plots) were generated with the clusterprofiler package v4.14.3 [74, 75] after running the *enrichKEGG* function on the respective DEGs. To compute the average expression of entire gene sets on a single-cell level, Seurat’s function *AddModuleScore* was used. For the stellate cells within the co-culture scRNA-seq data set, the module scores for different CAF subtypes were calculated, using the DEGs described by Toa et al. and Elyada et al. as input [39, 40]. The cells were then assigned a CAF subtype, based on the highest module score over all gene sets. Cells for which the highest module score was below the mean score across all cells were labeled as “non-assigned”.

### Velocity analysis

To investigate developmental trajectories within the time-series scRNA-seq data set, the Python package scVelo v0.3.2 [76] was used to determine cellular dynamics based on slicing kinetics. The processed Seurat object, including *KRAS*^*G12D*^-dependent grouping and UMAP embeddings, was therefore converted to an AnnData object with SeuratDisk (v 0.0.0.9021) and merged with the respective loom files. Data was then filtered and normalized using min_shared_counts = 20 and n_top_genes = 2000. First and second order moments were computed on the first 30 PCAs and 30 neighbors. Velocities were then estimated using the dynamical mode, and partition-based graph abstraction (PAGA) [77] maps were constructed on default parameters.

### ATAC-sequencing

PDLOs were harvested on day 3, day 5, and day 7 after the start of Dox treatment, including matched controls without Dox treatment. As KRAS expression at day 1 was considerably low (around 30% mCherry-positive cells) in comparison to day 3, 5, and 7 (Supplementary Fig. 1B), we did not include samples from day 1 for bulk ATAC-sequencing. 50,000 cells per sample were lysed and segmented using 2 × TD Tagment DNA Buffer containing TDE1 Tagment DNA Enzyme (Illumina), Digitonin (Promega), and Protease Inhibitor Cocktail (Roche). After 30 min of incubation at 37 °C, samples were purified using the MinElute Kit (Qiagen). The libraries were sequenced on a NovaSeq 6000 instrument (Illumina) following a 50-base-pair, paired-end recipe. Data quality control was done using the fastp tool and reads were aligned against GRCh38 with Bowtie2. Peaks were called with MACS2 and were used as input to create GRanges objects in R using GenomicRanges v1.50.2 [78]. Peaks were filtered to be present in at least 10 samples. Read counts were generated from bam files using the featureCounts function of the Rsubread package 2.12.3 [79]. DESeq2 v1.38.3 [80] was used to determine differentially accessible regions, which were then annotated using the ChIPseeker package.

### Proteome

PDLOs on day 25 after start of differentiation were re-seeded into 1196 microwell-chips as previously described [30]. On day 27, 3 µg/mL Dox was added and PDLOs were cultured on chip for another 7 days. For harvesting of samples, PDLOs were retrieved from chips through mechanical up- and down-pipetting using cold PBS and cells were lysed using RIPA buffer (Thermo Fisher Scientific). Tryptic digest was performed using 10 µg lysate and peptides were subjected to mass spectrometry on a Q-Exactive HF-X mass spectrometer (Thermo Scientific).

### Survival analysis of the TCGA-PAAD data set

In R, the TCGA-PAAD data set was downloaded using the TCGAbiolinks package v2.26.0 [81]. The data set was filtered to include only samples labeled as primary tumor and TPM values from RNA-seq were log transformed. The GSVA package was used to calculate gene set variation scores of each sample for the top 50 DEGs of KRAS^high^ or KRAS^intermediate^ compared to KRAS^low^. Samples were then grouped, where scores greater than 0.2 were considered as high enrichment while scores below 0.2 were considered as low gene enrichment. Survival curves were generated using the ggsurvplot of the survminer package v0.4.9.

### Reprocessing of published scRNA-seq data sets

The scRNA-seq data from Peng et al*. *[33], including 24 PDAC and 11 healthy samples, was downloaded from the Genome Sequence Archive (PRJCA001063). Reprocessing was done in R using the Seurat package [64–66], where the count matrix was filtered to include only cells with less than 10% mitochondrial genes and less than 8000 genes overall. Raw counts were normalized and log transformed. The top 2000 variable features were used to scale data and perform PCA on the first 50 PCs. Nearest neighbor and UMAP construction were performed using the first 20 dimensions. Cell type annotation was adopted from the information provided by the authors. For downstream analysis, the generated AnnData object was converted to a Seurat object using SeuratDisk v 0.0.0.9021.

The scRNA-seq data set from Carpenter et al. [19], which included pancreatic tissue of healthy donors with and without PanIN lesions as well as tumor tissue, was downloaded from the NIH Gene Expression Omnibus database (GSE229413). Data processing was performed according to the authors' analysis pipeline. Therefore, using Seurat, data was filtered to exclude cells with fewer than 200 unique genes and more than 15% mitochondrial counts. Fibroblasts were then extracted, and the individual samples were log-normalized, followed by anchor-based integration using the reciprocal PCA. Nearest-neighbor graph construction and UMAP dimensional reduction was done using the first 30 and 15 dimensions, respectively.

### Integration of PDLO scRNA-seq data set with a published PDAC scRNA-seq data set[33]

To create a virtual cancer space in silico, the Seurat objects of the time-series PDLO scRNA-seq and the PDAC scRNA-seq from Peng et al. [33] were merged and further processed using the Seurat package v4.0.6 [63, 64, 66]. Using the SplitObject function, a list containing both data sets separately was created, allowing normalization and finding variable features (*n* = 4000) individually on each data set. This list was then used as input to select integration features and to find integration anchors. The data sets could then be integrated based on the previously defined anchor set. The integration was then used as default assay and the top 500 variable features were used to perform PCA. The first 15 nearest neighbors were used and UMAP construction was applied with the first 30 dimensions.

### Grouping of PDAC samples based on deep learning classifiers

To group the samples of the PDAC scRNA-seq data set [33] based on the KRAS-PDLO deep learning classifiers, the data set was first filtered to include only ductal cells, on which normalization and variance stabilization were then performed using sctransform v0.4.1 [69, 70]. The count matrix was then extracted and restricted to the genes of the machine learning classifiers. Following, distance matrix computation (dist), hierarchical clustering (hclust) and kmeans grouping (cutree, k = 3) was computed with the R package stats v4.2.2. Based on the KRAS expression, these three groups were then assigned to “low”, “intermediate” or “high”, reflecting the KRAS clusters from the PDLO scRNA-seq data set.

### Cell–cell communication analysis

Cell–cell communication analysis was performed on the KRAS-grouped PDAC scRNA-seq data set and the integrated PDLO-PDAC scRNA-seq data set independently using the R package CellChat^65^ v2.1.2. For the grouped PDAC data set (Fig. 2H-I), the Seurat object was subsetted based on the KRAS groups, thereby creating three new Seurat objects, including cells of either the KRAS high, KRAS intermediate, or KRAS low samples. The subsetted Seurat objects were again normalized using the SCTransform v0.4.1 [69, 70] function. For the analysis of the integrated PDLO-PDAC data set, all cells were used without further subsetting. The respective Seurat objects were then used as input to create CellChat objects, on which communication probability and aggregated cell–cell communication networks were calculated as described in the package vignette using default parameters.

To investigate potential cell–cell communication between our time-series PDLOs and healthy, non-cancerous microenvironment cell types, the pre-processed scRNA-seq data set from Carpenter et al. was filtered to only include T cells, macrophages, epithelial cells, endothelial cells, fibroblasts, B cells, and natural killer cells (NK) from the healthy donors. The data set was then merged with the PDLOs from the time-series scRNA-seq and the merged raw count matrix was normalized using sctransform v0.4.1. Communication probabilities and aggregated cell–cell communication networks were then calculated using CellChat [82] v2.1.2. on default parameters.

To analyze ligand-receptor interactions within the PDLO co-cultures, the monoculture conditions were excluded and remaining cells were normalized with sctransform. Gene expression values were then smoothend based on the human protein–protein interaction (PPI) network before communication probabilities were calculated using the truncated mean, followed by the computation of aggregated cell–cell communication networks. The same procedure was applied to the treatment conditions separately (Dox-treated, Dox-treated + infliximab, and untreated). The individual CellChat objects were then compared using the *mergeCellChat* function, allowing the pairwise comparison of altered signaling patterns.

### Isolation of T cells from healthy donors

Buffy coats from healthy donors were purchased from the German Red Cross blood bank. The donors gave written consent to process the blood samples for research purposes, and the Ethics commission of Ulm University approved the study (#460/23). For T cell perfusion of tumor-chip models, whole blood was collected from healthy donors after informed consent as approved by the Ethical Committee of the Eberhard Karls University Tübingen (No. 495/2018-BO02). First, peripheral blood mononuclear cells (PBMCs) were isolated from buffy coats by density gradient centrifugation. Therefore, buffy coats were diluted 1:1 with DPBS and carefully layered onto Ficoll-PaqueTM PLUS. After centrifugation at 800 × g for 20 min, PBMCs could be collected and were washed three times in DPBS. PBMCs were then either directly used for T cell isolation or cryo-preserved in heat-inactivated FBS + 10% DMSO. For T cell isolation, the Dynabeads™ Untouched™ Human T Cells Kit (Invitrogen) or the Pan T Cell Isolation Kit, human (Miltenyi) was used following the manufacturer’s protocol, allowing negative selection by MACS. In brief, PBMCs were mixed with the provided antibody cocktail, followed by incubation with the magnetic beads. Using a magnet, purified T cells could then be retrieved from the supernatant. For activation, T cells were seeded on plates pre-coated with anti-CD3E and anti-CD28 antibody (BD) or together with 1:100 diluted T Cell TransAct™, human (Miltenyi), and cultured in RPMI + 10% heat-inactivated FBS overnight or in X-Vivo 15 Medium (Lonza) with 10% FetalClone II Serum (FCS) (HyClone, Cytiva) and 1% Pen/Strep (10,000 U/mL, Pan Biotech) for 4 days.

### Co-culture of matrix-embedded organoids and T cells

In order to investigate T cell infiltration, the INTERaction with Organoid-in-MatriX ("InterOMaX") platform was used as previously described [34, 83, 84]. Therefore, agarose chips (2% agarose in PBS) were prepared using the 81-microwell casts from Microtissues® (Sigma-Aldrich). PDLOs were harvested and dissociated into single cells. 50,000 cells were seeded per chip, allowing organoid formation with one organoid per microwell for 24 h before starting Dox treatment (3 µg/mL). After 4 days of Dox treatment, a mixture of collagen type 1 (Merck) and Matrigel (7:3) was added, and the matrix patches including the embedded organoids were transferred to chamber slides. T cells isolated from healthy donor PBMCs were activated by exposure on tissue culture plates coated with anti-CD3E/-CD28 overnight (anti-CD3E: #555,329, anti-CD28: #555,725, both from BD), then stained with 1 µM CellTracker™ Green CMFDA (Invitrogen). Stained T cells were added to the embedded organoids with 5 × 10^5^ T cells per chamber (10:1 T cell to organoid cell ratio). After 72 h, T cell infiltration into organoids was analyzed by fluorescence microscopy (BZ-X Series, Keyence) and quantified in an automated manner using the ImageJ Macro Watershed Segmentation technique [34].

### Co-culture of PDLOs and T cells using a cancer-on-chip platform

To investigate T cell infiltration under physiological conditions, a cancer-on-chip platform was used as previously described [35]. Therefore, PDLOs were resolved in pure GFR Matrigel, seeded in PDMS chips with approximately 30 organoids per chip and treated with 3 µg/mL Dox for 4 days under perfusion. Subsequently, T cells, isolated from healthy donors and stained with 10 µM of Cell Tracker CMFDA (Invitrogen) in PBS with Calcium and Magnesium (Pan Biotech), were added to the media channel, with 200,000 T cells/chip and a flow rate of 20 µL/h. Chips were imaged on day 1 and day 3 after T cell perfusion using a fluorescence microscope (Axio Observer 7, Carl Zeiss) and T cell infiltration was quantified with ImageJ-Fiji software.

### Generation of PDLO-conditioned media

PDLOs on day 27 after start of differentiation were harvested and 100,000 cells were re-seeded per 12-well plate, precoated with an Ultimatrix layer (300 µL/well). After 4 days, allowing reformation of organoid structures, 3 µg/mL Dox was added for another 6 days. Media was then completely removed and the Ultimatrix layer containing the PDLOs was washed with PBS for 5 min. Next, 1 mL blank DMEM (Gibco) was added per well for 24 h. The conditioned media was then collected, filtered through a 0.2-µm filter to exclude cell debris, and stored at −20°C until further use.

### Transwell migration assay

Activated T cells were resuspended in blank FBS-free DMEM and seeded into Transwell® 8.0-µm Pore Polycarbonate Membrane Inserts (Corning) with 500,000 cells/insert. Inserts were then transferred to a 24-well plate containing 500 µL of conditioned media and T cells were allowed to migrate for 2 h at 37°C. Cells from upper chamber (insert) and lower chamber (well) were then collected separately and counted. To determine the amount of migrated cells, the cell count from the lower chamber was normalized to the total number of cells within each well.

### Immune panel flow cytometry analysis of T cells

Activated T cells were cultured either in PDLO-conditioned media or indirectly co-cultured with PDLOs in cell culture inserts (Thermo Fisher) for 72 h before T cells were used for flow cytometric analysis. For surface staining, cell pellets were resuspended in 100 μL of PBS with 0.5% BSA containing respective fluorophore-conjugated antibodies and incubated for 30 min at 4 °C protected from light. Cells were then washed with PBS with 0.5% BSA, centrifuged at 400 × g at room temperature and resuspended in 300 μL PBS with 0.5% BSA for flow cytometric analysis. Data were acquired on a Cytek Aurora spectral flow cytometer and analyzed with FlowJo software v10.81.

### Antibody panel

**Table:** 

Target	Conjugate	Cat.Nr	Company
Viability	ViaDye Red Fixable Viability Dye	SKU R7-60,008	Cytek
CD3	Pac Orange	CD0330	Life Technologies
CD4	AF700	56–0048–82	Invitrogen
CD8	BV650	344,729	Biolegend
CD25	FITC	356,106	Biolegend
PD-1	PE	12–2799-42	Invitrogen
CD127	PE-Cy5	351,324	Biolegend
HLA-DR	APC-H7	561,358	BD Biosciences

### Live imaging of PDLO-PaSC co-cultures

PDLOs were re-seeded in a 1:4 ratio on 96-well plates precoated with an Ultimatrix layer (50 µL/well) and treated with Dox (3 µg/mL) for 4 days before 50,000 GFP-labeled PaSCs were added. The co-culture was then monitored using the Incucyte® S3 live-cell imaging system (Sartorius), applying the spheroid multi module, imaging every 12 h. Using the Incucyte® software, images were quantified regarding the total green area by top-hat segmentation.

### Indirect co-culture of PDLOs and PaSCs

PDLOs were treated with Dox for 4 days, followed by re-seeding in 50-µL GFR Matrigel domes at a 1:3 ratio on 12-well plates. In parallel, PaSCs were harvested using TrypLE. After solidification of the PDLO dome, PaSCs were seeded in a 50-µL dome with 70,000 cells/dome within the same well. The cells were co-cultured for 72 h before each dome was harvested separately by mechanical scraping with a pipette tip. Cells were then washed once with PBS and further processed for downstream analysis.

### Macrophage differentiation and chemotaxis assay

#### Isolation and differentiation of human monocytes

Human monocytes were isolated from peripheral blood of three healthy donors by positive selection of CD14^+^ cells using magnetic beads (MACS; Miltenyi Biotec, Bergisch-Gladbach, Germany) according to the manufacturer’s instructions. Monocytes were differentiated into macrophages following a previously published protocol (PMID 31573680). After differentiation, macrophages were harvested by gentle non-enzymatic detachment using Cellstripper (Corning).

#### Plate preparation and assay setup

The upper and lower chambers of the Incucyte® Clearview 96-Well Plate (upper) and the corresponding Clearview Reservoir Plate (lower) were coated with growth factor–reduced (GFR) Matrigel (Corning) at a protein concentration of 50 µg/ml diluted in DMEM. The top chamber was coated with 20 µl, and the bottom chamber with 150 µl of Matrigel solution, followed by polymerization for 1 h at 37 °C and 5% CO₂. After polymerization, excess medium was removed from the top chambers. Macrophages were counted and seeded at a density of 2.500 viable cells per well in 60 µl of assay medium (RPMI 1640 supplemented with 0.5% FCS and 1% Penicillin/Streptomycin). Cells were allowed to settle for 45 min at room temperature. Meanwhile, bottom layer wells were prepared with the respective conditioned media or control medium. Duplicate wells were prepared for each condition and donor. After the settling period, the top plate was assembled onto the bottom plate and incubated for 15 min at 37 °C to equilibrate. Condensed water on the lid was carefully removed before initiating the imaging program. Real-time imaging was performed every 15 min for 48 h using a Sartorius Incucyte® SX5 Live-Cell Analysis System. Quantitative analysis of macrophage migration was carried out using the Incucyte® Chemotaxis Analysis Software Module (Sartorius).

### In-well immunofluorescence staining of bi-cultures

PDLOs were seeded in 8-well slides (Ibidi), pre-layered with GFR Matrigel, and treated with 3 µg/ml Dox for 4 days. T cells (isolated and activated as described above) were then incubated with CellTracker™ Blue CMAC (Invitrogen) for 30 min at 37 °C before adding them to the PDLOs in a ratio of 1:10. After 3 days of co-culture with PDLOs growing on the GFR Matrigel and T-cells floating in the medium suspension, cells were fixed with 4% paraformaldehyde (PFA) and 100 mM sucrose for 30 min at RT. For permeabilization and blocking, 5% normal donkey serum and 0.1% Triton-X in PBS (blocking buffer) was added to the wells. Co-staining with primary antibodies was performed overnight at 4 °C, targeting the HA-Tag of our *KRAS*^*G12D*^ construct (#3724, Cell Signaling) and KRT19 (#M0888, Dako). Secondary antibodies were added for 1 h at room temperature using Alexa Fluor™ 568 anti-rabbit (Invitrogen) and Alexa Fluor™ 488 anti-mouse (Invitrogen), both 1:500. Draq5 (5µM) was used to counterstain the nuclei. The slides were then imaged with the Leica TCS SP8 confocal microscope.

### Enzyme-Linked Immunosorbent Assay (ELISA)

To determine the secreted TNFα levels of our PDLOs, conditioned medium was generated as described above. Using Pierce Protein Concentrators (3 MWCO, Thermo), conditioned medium was concentrated, reducing the volume from 1 ml to 300 µl by centrifugation at 4000 × g for 20 min. For detection and quantification of TNFα, the Human TNFα-ELISA Kit (Invitrogen) was used, following the manufacturer’s protocol. Concentrations were then calculated by interpolating the absorbance values with the standard curve.

### Cell cycle analysis (EdU staining)

For cell cycle analysis, 10 µM EdU was added to the PDLOs for 4 h at 37°C. Cells were then harvested as described above and fixed on ice with 4% paraformaldehyde (PFA) and 100 mM sucrose for 20 min on ice. For permeabilization, samples were incubated in 5% normal donkey serum and 0.1% Triton-X in PBS and incubated overnight with primary anti-HA-Tag antibody (Cell Signaling #3724S, 1:1000). Secondary antibody (Alexa Fluor™ 488 anti-rabbit, Invitrogen, 1:500) was added for 90 min on ice after washing the samples in 2% normal donkey serum and 0.1% Triton-X in PBS. Samples were again washed and EdU was stained using the Click-iT EdU Alexa Fluor 647 Assay Kit (Life Technologies). Samples were then measured with the Attune™ NxT (Thermo Fisher Scientific) flow cytometer and analysis performed using the FlowJo software.

### Quantitative real-time PCR

For RNA isolation, the Gene-JET RNA Purification Kit (Thermo) or the Monarch® Total RNA Miniprep Kit (New England Biolabs) was used following manufacturer's protocol. Subsequently, cDNA synthesis was performed using the iScript cDNA Synthesis Kit (Bio-Rad). The generated cDNA was used as input for quantitative real-time PCR (qPCR), in combination with the GreenMasterMix (2x) No ROX (Genaxxon) and respective primers. Samples were measured on the Rotor-Gene Q (Qiagen) or the QuantStudio 3 Real-Time PCR System (Thermo) and Ct values were then normalized to the house-keeping gene Glycerinaldehyd-3-phosphat-Dehydrogenase (*GAPDH*).

The following primes were used:

- *ACTA2* (for_5´-AGATCAAGATCATTGCCCC-3´, rev_5´-TTCATCGTATTC CTGTTTGC-3´, Biomers),
- *CXCL2* (for_5´-TGCAGGGAATCTCAAG-3´, rev_5´-TGAGACAAGCTGCCCA-3´, Biomers),
- *CTGF* (for_5´-CCTGGTCCAGACCACAGAGT-3´, rev_5´-TGGAGATTTTGGGAGTACGG-3´, Biomers),
- *GAPDH* (for_5´-TCGGAGTCAACGGATTTG-3´, 5´-CAACAATATCCACTTTACCAGAG-3´, Biomers),
- *IL-6* (for_5´-GCAGAAAAAGGCAAAGAATC-3´, rev_5´-CTACATTTGCCGAAGAGC-3´, Biomers),
- *POSTN* (for_5´-TGTCACTGTTAATTGTGCTC-3´, rev_5´-CTGCTCTAAAAGATGAAAGGTC-3´, Biomers)
- *IL12B (*for_5’-TGCCCAGAGCAAGATGTGTC-3’rev_5’-CGAGGGGAGATGCCAGAAAA-3’, Biomers),
- *CXCL9* (for_5’-GATTGGAGTGCAAGGAACCC-3’rev_5’-TAGTCCCTTGGTTGGTGCTG-3’, Biomers),
- *CXCL10* (for_5’-AGTGGCATTCAAGGAGTACCT-3’, rev_5’-CGTGGACAAAATTGGCTTGC-3’, Biomers),
- *IL6* (for_5’-CCTTCTCCACAAGCGCCTTC-3’, rev_5’-GGAAGGCAGCAGGCAACA-3’, Biomers),
- *CD44* (for_5’-ACAACCACAAGGATGACTGATGT-3’, rev_5’-GTGAATGAGGGGAGGGTGTG-3’, Biomers),
- *MMP9* (for_5’-GGCGCTCATGTACCCTATGT-3’, rev_5’-TTCAGGGCGAGGACCATAGA-3’, Biomers),
- *CD163* (for_5’-GCCATTCTGAGCCACACTGA-3’,rev_5’-AGGTATCTTAAAGGCTGAACTCACT-3’, Biomers),
- *HMBS* (Hs_HMBS_1_SG QuantiTect QT00014462, Qiagen)

### Taqman assay

For copy number analysis, gDNA was isolated from hESC, PDO and cancer cell lines using the DN easy Blood and Tissue Kits for DNA Isolation (Qiagen) following manufacturer's protocol. The monoallelic BU3 NGST-TetOn:KRASG12D iPSC line [91] was included as an additional control. For each assay, the gDNA was diluted to an equal concentration between 3–5 ng/µl in all samples. The copy number of KRAS was measured by using respective probes and primers. For the endogenous KRAS locus, a commercial KRAS FAM target probe assay mix was used (Hs02739788_cn, Thermo Fisher Scientific) which recognizes a region within the 3’UTR of exon 5. For measuring the total KRAS copy number, a self‐designed KRAS exon 3 FAM probe (5 ‘-/56-FAM/TCG ACA CAG/ZEN/CAG GTC AAG AAG AGT A/3tABkFQ/−3’, IDT) and its respective forward and reverse primers (for_5’GAAGCAAGTAGTAATTGATGGAGAA-3’, rev_5’-CCAGTCCTCATGTACTGGTC-3’, biomers) were used. As a reference for a biallelic copy number, a commercial probe assay mix for RNAseP that carries the VIC fluorophore (Thermo Fisher Scientific) was included in each reaction. 10 µl of each sample reaction was measured in a 96-well plate using the QuantStudio™ 3 Real-time PCR machine (Thermo Fisher Scientific). Ct values were then normalized to the RNAseP reference gene.

### Western blot

Cells were lysed in RIPA buffer (50 mM Tris pH 7.4 (AppliChem), 150 mM NaCl (Sigma), 1 mM EDTA (AppliChem), 1% NP40 (Fluka), 0.25% Sodium deoxycholate (Sigma), 0.1% SDS (Serva), 1 mM PMSF (AppliChem), 1 × phosphatase inhibitor, and 1 × EDTA-free protease inhibitor cocktail (cOmplete; both Roche)) for 30 min on ice. After centrifugation at 10,600 × g for 8 min to remove cell debris, protein concentrations were determined using the Pierce™ BCA Protein Assay Kit (Thermo). Equal amounts of total protein were then separated by SDS-PAGE (BioRad) and transferred to a PVDF membrane (Millipore), using the Transblot semidry transfer system (BioRad). To saturate unspecific binding sites, membranes were incubated in blocking solution (5% BSA in TBS buffer + 0.1% Tween20 (TBST)) for 1 h at RT before adding primary antibody diluted in blocking solution overnight at 4°C. Membranes were then washed three times in TBST and incubated with secondary antibody for 1 h at RT (anti-mouse-horseradish peroxidase (HRP) or anti-rabbit-HRP, GE Healthcare, 1:1000 in blocking solution). The SuperSignal West Dura Kit (Thermo) was used for protein detection and imaging of the membranes was performed with the Chemiluminescence Imaging – Fusion SL system (VILBER). ImageJ software was used for quantification. The following primary antibodies were used: anti-ERK1/2 (Cell Signaling Cat#9102, 1:1000), anti-phospho-ERK1/2 (Cell Signaling Cat#4377, 1:1000), anti-GAPDH (Merck, #G9545, 1:1000).

### Flow cytometry

Adherent cells grown in monolayer, such as PPs and PaSCs, were harvested using TrypLE, while PDLOs were collected using collagenase/dispase (Roche) and Accutase solution (Merck). Cells were then fixed in 4% paraformaldehyde (PFA) and 100 mM sucrose for 20 min on ice. After washing with PBS, samples were incubated in 5% normal donkey serum and 0.1% Triton-X in PBS (FC buffer) for permeabilization and blocking. Primary antibody, diluted in FC buffer, was then added and samples were incubated overnight at 4°C. Samples were then washed three times in 2% normal donkey serum and 0.1% Triton-X in PBS (wash buffer), before adding secondary antibody diluted in FC buffer for 90 min on ice. For the use of conjugated primary antibodies, incubation followed directly after blocking. Samples were then washed again three times in wash buffer before measuring samples with an Attune™ NxT (Thermo Fisher Scientific) flow cytometer. Analysis was performed using the FlowJo software. The following antibodies were used: anti-aSMA-APC (R&D Systems, IC1420A, 1:20), anti-IL6-PE-Cy7 (Thermo Scientific, 25–7069-42, 1:10), anti-PDX1 (R&D Cat#AF2419, 1:500), anti-PDX1-PE (BD Cat#562,161, 1:35), anti-NKX6-1 (DSHB Cat#F55A12, 1:150), anti-NKX6-1-APC (BD Cat#563,338, 1:35).

### CyTOF analysis of murine fibroblasts

Antibodies were purchased in a carrier protein-free form were labelled with the indicated heavy-metal tag using Maxpar X8 or MCP9 Antibody Conjugation Kit (Standard Biotools) as per manufacturer’s recommendations. Mouse primary pancreatic fibroblasts (mPAFs) were isolated from healthy wild-type mouse pancreata. In detail, minced pancreata were disaggregated in DMEM supplemented with 20% FBS, 1 mg/mL collagenase P (#1,121,386,500, Merk) and 50 µg/mL DNAse I (#10,104,159,001, Sigma-Aldrich), at 37 °C with shaking. Disaggregated tissue was cultured and expanded in DMEM supplemented with 20% FBS. Epithelial, endothelial and immune cells were depleted by MACS® separation (Miltenyi Biotec) using magnetic MicroBeads coated with EpCAM, CD31 and CD45 antibodies, respectively. A total of 3 × 10^5^ mPAFs were seeded into 11 TC-treated cell culture flasks and incubated overnight in DMEM supplemented with 20% FBS. The following day, the media in each culture flask was replaced with 10 mL of fresh DMEM supplemented with 20% FBS containing the appropriate ligand. After 72 h, stimulated mPAFs, were washed twice with PBS and lifted with Accutase, filtered through a 70-µm strainer. The cell pellets were resuspended in CSM3 (500 mL PBS with 2.5 g BSA, 2.5 mL FBS and 100 mg DNAse1 (200 µg/mL), sterile filtered through 0.1-µm filter cup), and counted, 20 μL of 100 U/mL heparin sodium salt (Sigma Aldrich, H3393) solution in PBS and 1 μL Fc block (BD Biosciences, 558,636) was added and incubated on ice for 5 min. Cell pellets were resuspended in a master mix of extracellular targeting metal-conjugated antibodies diluted in 50 μL of CSM3 and incubated on ice for 45 min. Viability staining used 300 nM solution of 167Er-Mal-DOTA in PBS 5 mM EDTA [85] for 15 min on ice. Cells were fixed for 30 min with 1 mL of 1 × FOXP3 Fixation Buffer (Thermo Fisher, 00–5523-00) at RT, followed by cell permeabilization with 2 mL of 1 × FOXP3 Permeabilization buffer. Cells were resuspended in 1 mL of 10% v/v DMSO (Sigma Aldrich, D2650) in CSM1 (Cell Staining Buffer – Intracellular), consisting of 5 mg/mL BSA and 0.2 mg/mL sodium azide in PBS, and frozen at −80°C. Samples were barcoded using the Cell-ID 20-plex Pd Barcoding Kit (Standard Biotools, 201,060) following manufacturer’s instructions. Barcoded samples were pooled in FOXP3 Permeabilization Buffer. For each sample included in the pooled sample, 10 μL of 100 U/mL heparin sodium salt in PBS and 0.5 μL of Fc block was added and the sample mixed by gently rocking for 5 min at RT. Pelleted cells were resuspended in a master mix of intracellular targeting, metal-conjugated antibodies diluted in CSM1 was added and incubated for 45 min. Cells were fixed and DNA staining with 3 mL of 4% Paraformaldehyde (Thermo Fisher, 28,908) in PBS and 1 µL 500 µM 103Rh (Standard Biotools, 201103 A) overnight at 4°C. After washing, the cell pellets were resuspended at a concentration of 1 × 10^6^ cells/mL in 15% EQ Four Element Calibration Beads (Fluidigm, 201,078), filtered twice through 70-μm Filcons (BD Biosciences, 340,633) and acquired on a Helios Mass Cytometer (Fluidigm) using a Super Sampler.

FCS files were normalised for signal-drift during the acquisition run using the in-built Helios normalisation tool (Fluidigm). Individual sample events were debarcoded using the R package “Premessa”, with a Mahalanobis distance of 15 and a minimum barcode separation of 0.3. Individual sample FCS files were uploaded to the cloud-based cytometry platform Cytobank (https://www.cytobank.org, Beckmann Coulter) and checked for consistent signal across the entire acquisition period and correct deconvolution. As per standard methods, live cell events were cleaned up based on Gaussian parameters, selected based on DNA-103Rh positivity and mal-DOTA-167Er negativity. Cell doublets and aggregates were removed based on event length. All cell events were exported and analysed using CATALYST 1.30.0).

**Table:** 

Mesenchymal stroma panel—extracellular					
Antibody target	**Metal**	**Clone**	**Pre-conjugated/custom**	**Supplier**	**Identifier**
Fc block		2.4G2		BD Biosciences	558,636
CD105 (biotinyl.)		MJ7/18		Biolegend	120,404
CD44	089Y	IM7	Custom	Biolegend	103,002
Sca1	111Cd	D7	Custom	Biolegend	108,102
EpCAM	113In	G8.8	Custom	Biolegend	118,202
Ly6C	114Cd	HK14	Custom	Biolegend	128,002
CD86	139La	GL-1	Custom	Biolegend	105,002
MCAM	141Pr	ME-9F1	Pre-conjugated	Fluidigm	3141016B
ITGA5	142Nd	5H10-27	Custom	Biolegend	103,801
CD87	144Nd	109,801	Custom	Thermo Fisher	MA5-23,853
ITGA1	145Nd	Ha31/8	Custom	BD Biosciences	555,001
ITGAV	146Nd	RMV-7	Custom	Biolegend	104,102
ITGA2	147Sm	HMa2	Custom	Biolegend	103,501
PDGFRA	148Nd	APA5	Pre-conjugated	Fluidigm	3148018B
PDPN	149Sm	8.1.1	Custom	Biolegend	127,402
CD24	150Nd	M1/69	Pre-conjugated	Fluidigm	3150009B
PDGFRB	151Eu	APB5	Pre-conjugated	Fluidigm	3151017B
FAP	152Sm	73.3	Custom	Merck	MABC1145
MSLN	153Eu	B35	Custom	MBL	D233-3
CD63	155Gd	NVG-2	Custom	Biolegend	143,902
CD73	156Gd	TY/11.8	Custom	Biolegend	127,202
CD26	160Gd	H194-112	Custom	Biolegend	137,802
ITGB3 (CD61)	161Dy	HMB3-1	Custom	Biolegend	104,302
CD34	162Dy	MEC14.7	Custom	Biolegend	119,302
ITGA6	164Dy	G0H3	Custom	Biolegend	313,602
Biotin	165Ho	1D4-C5	Pre-conjugated	Fluidigm	3165012B
CD14	166Er	ME20.4	Custom	Biolegend	123,302
CD80	168Er	16-10A1	Custom	Biolegend	104,702
CD31	170Er	MEC13.3	Custom	Biolegend	102,502
CD38	171Yb	90	Pre-conjugated	Fluidigm	3171007B
ITGB1	172Yb	HMB1-1	Custom	Biolegend	102,202
VCAM1	173Yb	MVCAM.A	Custom	Biolegend	105,702
CD45	175Lu	30-F11	Pre-conjugated	Fluidigm	3175010B
CD90	176Yb	G7	Custom	Biolegend	105,202
MHCI (H2Db)	194Pt	28–14-8	Custom	Biolegend	114,502
ICAM1	195Pt	YN/1.7.4	Custom	Biolegend	116,102
CD39	196Pt	24DMS1	Custom	Thermo Fisher	14–0391-82
CD74	198Pt	In1/CD74	Custom	Biolegend	151,002
MHCII	209Bi	M5/114.15.2	Pre-conjugated	Fluidigm	3209006B
Mesenchymal stroma panel—Intracellular					
Antibody target	**Metal**	**Clone**	**Pre-conjugated/custom**	**Supplier**	**Identifier**
Fc block		2.4G2	-	BD Biosciences	558,636
Cytokeratin-7	115In	RCK105	Custom	Abcam	Ab9021
Pan-cytokeratin	116Cd	C11	Custom	Biolegend	628,602
POSTN	143Nd	345,613	Custom	R & D	MAB3548
PDPN	149Sm	8.1.1	Custom	Biolegend	127,402
FAP	152Sm	73.3	Custom	Merck	MABC1145
RFP	153Eu	8E5.G7	Custom	Rockland Inc	200–301–379
MSLN	153Eu	B35	Custom	MBL	D233-3
VIM	154Sm	D21H3	Pre-conjugated	Fluidigm	3154014A
CD63	155Gd	NVG-2	Custom	Biolegend	143,902
DES	158Gd	Y66	Custom	Abcam	ab271829
aSMA	159 Tb	1A4	Custom	Abcam	ab240654
Cleaved caspase-3 (CC3)	163Dy	D3E9	Custom	Cell Signaling Technology	9579
Ki67 (169 version, new)	169Tm	SolA15	Custom	Thermo Fisher	14–5698-82
Intracellular Collagen IV	174Yb	pAb ab6586	Custom	Abcam	ab6586
CD74 (198 version, new)	198Pt	In1/CD74	Custom	Biolegend	151,002

### Analysis of cyst fluid from IPMN patients

#### Patients enrolment

All consecutive patients with resected, histologically confirmed IPMNs prospectively enrolled under the “Endocrine, Metabolic, Inflammatory Biomarkers to Identify High grade Dysplasia/Invasive Carcinoma in Patients With IPMN of the Pancreas (EMI-IPMN)” study (NCT06706700) and “Integration of Multiomics Markers for Invasive IPMNs Identification Through the Set-up of the INvasive Cyst bIomarkers Detection (INCITE) Consortium” study (NCT06694792) with undiluted cyst fluid were analysed. Patients were enrolled at the Pancreatic Surgery and Transplantation department at IRCCS San Raffaele Hospital in Milan, Italy, between 1 st July 2020 and 5th January 2025 for the patients in the EMI-IPMN cohort, and from the 6th January 2025 to 1 st August 2025 for the patients in the INCITE study. Patients enrolled in the study underwent surgical resection in accordance with the 2017th international guidelines [86].

#### Pathological examination

Histologic examination of the surgical specimen was based on the 2019 WHO Classification of Tumours [87]. Dysplasia was classified in low grade (LGD) and high grade (HGD), using the classification system from the 2015 Baltimore consensus meeting for neoplastic precursor lesions of the pancreas [88]. TNM classification is based on the 8th edition of the American Joint Committee on Cancer (AJCC) staging system for pancreatic cancer [89].

#### Samples collection

Cyst fluid (CF) from the IPMN was drawn from the surgical specimen before it was sent for pathological examination. In case of branch duct IPMN it was drawn from the cyst after its manual identification, in case of main duct involvement the fluid was drawn from the main duct after the sectioning of the pancreas. The collection happened in the operating room, in sterile conditions, using a 25G sterile syringe. The CF was immediately refrigerated and processed within 60 min from the collection. To avoid deterioration from the pancreatic juices to each sample were added DPP4 inhibitor (concentration 1:100, Merck, cat. DPP4-010), Pefabloc (conc. 1mg/mL, Merck, cat. 101,500-500MG), Proteinase inhibitor cocktail (conc. 1:1000, Merck, cat. P2714-1BTL) and Aprotinin (conc. 500 KIU/ml, Merck, cat. A6279-10ML). After processing the samples were cryopreserved in liquid nitrogen until the time of analysis.

#### Cyst fluid analysis

CF was analysed using the MILLIPLEX® human metabolic hormone panel v3 kit (MERCK-MILLIPORE, cat. HMH3-34K) according to the protocol provided by the manufacturer. The output was read with the MAGPIX (Thermofisher) or Luminex 200 instrument (Thermofisher). The levels of TNFa were compared between patients with different grades of dysplasia using Mann Whitney test. The values are reported as median and interquartile range (IQR). Statistical analysis were performed using GraphPad Prism version 9.5.0 (2022).

### *RNAscope in situ hybridization with combined immunofluorescence*

For RNAscope experiments, two sample types were analyzed: (i) Ptf1a-Cre;Kras^LSL−G12D/+^ (KC) mice at 10, 20, and 36 weeks (*n* = 3 per timepoint) and (ii) 6 human pancreatic tissues from the Department of Pathology, Ulm University, with PanIN-comprising pancreata from resected PDAC samples.

Sections were deparaffinized in xylene, rehydrated through graded ethanol, and air-dried at 60 °C. Pre-treatment included incubation with RNAscope® Hydrogen Peroxide (10 min, RT), heat-induced target retrieval in 1 × Target Retrieval Reagent (30 min, 99 °C, steamer), and Protease Plus digestion (30 min, 40 °C) in a humidified chamber. Species-specific probes were hybridized for 2 h at 40 °C in a temperature-controlled incubator: RNAscope™ Probe – Mm-TNF-α (Mus musculus, Cat. 311,081) was used for murine tissue and RNAscope™ Probe – Hs-TNF-α (Homo sapiens, Cat. 310,421) for human sections. Slides were washed twice with 1 × Wash Buffer and subjected to the standard AMP 1–3 amplification sequence. Signal detection was performed with Opal™ 520 (C1 channel; Akoya Biosciences) diluted 1:3000 in TSA buffer. After RNAscope detection, immunofluorescent staining for E-Cadherin (CDH1) (Cell Signaling Technology, #3195, clone 24E10) was performed overnight at 4 °C, followed by Alexa Fluor™ 568 anti-mouse secondary antibody (Invitrogen, 1:500, 90 min, RT, dark). Slides were counterstained with DAPI (final concentration: 50 ng/mL) and mounted in Fluoromount G (Southern Biotech). Regions containing lesions of interest, were selected based on overview scans, and were then imaged on a VS200 slide scanner (Evident) using a 100 × objective and extended-focus imaging projection mode for subcellular resolution. Due to autofluorescence background in the FITC probe channel, background was reduced in ImageJ using a 30-pixel median filter for all images. For one mouse sample (week 10) and two human samples (PanIN low and PanIn low & high), background subtraction failed, so that artefacts could not be discriminated from actual TNFA spots. These samples were excluded from quantification/analysis. Since, relatively few PanIN lesion were found on the investigated human tissue samples, TNFA spots were only quantified for the murine images. Quantitative analysis was carried out in QuPath (v0.5). First, regions of interest (ROIs), constituting only epithelial cells of the respective lesion type of, were manually defined. E-Cadherin–positive epithelial cells were then determined using DAPI-based cell detection, and cytoplasm expansion set to 3µm followed by a binary E-Cadherin cytoplasm mean single measurement classifier. TNF-α probe spots were finally called by subcellular detection analysis using a mean threshold classifier combined with a size filter (0.2–2 µm^2^). Quantitative cellular metrics were region-wise exported and aggregated using R (v4.4.3) [90].

### Statistical analysis

Data from in vitro experiments were analyzed and visualized using GraphPad Prism. Statistical testing was performed as indicated. Statistical significance is displayed as *p* < 0.05: *, *p* < 0.01: **, *p* < 0.001: ***, *p* < 0.0001: ****.

## Supplementary Information

Supplementary Material 1: Supplementary Figure 1. (A) Brightfield images of PDLOs ± Dox treatment. Scale bar = 250 µm. (B) Flow cytometry quantifying mCherry reporter expression after Dox treatment. (C) Dot plot showing expression of cell type markers in PDLO scRNA-seq data set. (D) *KRAS* copy numbers for hESC lines, PDAC lines and patient-derived organoids (PDOs) using probes targeting either the 3’ untranslated region (3’UTR) of the endogenous locus or exon 3, which detects the respective sequence in the endogenous locus and in the inserted piggyBac KRAS^G12D^ construct. Copy numbers are shown relative to the respective KRAS wild-type cell lines (parental hESCs, BxPC3 and PDO#38188). BU3 = monoallelic BU3 NGST-TetOn:KRASG12D line^[92]^. (E) Scatter plot showing correlation between KRAS^G12D^ expression and module scores of different pathways over the time of Dox treatment within the scRNA-seq data set. R = Pearson’s correlation coefficient. (F) Protein expression of phospho-ERK1/2 and ERK1/2 in PDLOs after indicated time-points of Dox treatment, in PDAC lines and PDOs. For quantification, phospho-ERK1/2 levels were normalized to ERK1/2 levels for each sample. n = 3 for KRAS-PDLOs, n = 2 for parental PDLOs and n = 1 for PDAC lines and PDOs. (G) Cell cycle analysis of PDLOs after indicated time-points of Dox treatment assessed by EdU quantification via flow cytometry. n = 4. (H) PCA plot demonstrating variance of chromatin accessibility between ATAC-seq samples. (I) Heatmap showing mean gene expression of TFs, overlapping in ATAC- and scRNA-seq. Supplemental Figure 2. (A) Proportion of the KRAS clusters within the individual time points of the scRNA-seq analysis. (B) Expression of the top 10 DEGs from each KRAS cluster compared to all other clusters. (C) Overlapping and unique DEGs of KRAS^high^and KRAS^intermediate^ compared to the KRAS^low^ cluster with corresponding KEGG pathway analysis of unique DEGs. (D) Overall survival of TCGA-PAAD patients grouped based on KRAS cluster DEGs by gene set variance analysis (GSVA). (E) Pie charts showing the distribution of grouped GO terms from DEGs of KRAS^high^ and KRAS^intermediate^ compared to KRAS^low^. (F) Bar plot showing the relative abundance of proteins after 7 days of Dox treatment. Mean ± SD, n=1 in triplicates. Unpaired t-test **, p<0.01, ***, p<0.001; ****, p<0.0001. (G) Heatmap showing correlation coefficients of KRAS deep learning classifiers. (H) K-means grouping of healthy (N) and PDAC samples (T) from Peng et al.^[33]^ based on KRAS deep learning classifiers. (I) Box plot showing *KRAS*expression of PDAC samples from Peng et al.^[33]^grouped by k-means based on KRAS deep learning classifiers. Wilcoxon test ***, p<0.0001. (J) Bar plots showing tumor differentiation (left), perineural invasion (middle), and vascular invasion (right) of PDAC samples from Peng et al. [33] grouped by k-means based on KRAS deep learning classifiers. Chi-square test **, p<0.01. (A) Fluorescence intensities of all markers after initial background subtraction on human PanIN lesions subjected to 24-plex immunofluorescence staining. (B) Percentage of viable, exhausted, and activated T cells after culturing in PDLO conditioned media for 72 h determined by flow cytometry. Mean ± SD, n=2. (C) Percentage of T cells migrating towards PDLO conditioned media derived from a control line without KRAS^G12D^ construct (parental PDLOs). Mean ± SEM of 3 independent experiments. Unpaired t-test ns, p>0.05. (D) Representative images of parental PDLOs co-cultured with GFP-labeled pancreatic stellate cells (PaSCs) and respective quantification of the total green area over time of live-cell imaging. Mean ± SEM, n=3. Unpaired t-test ***, p<0.001; ****, p<0.0001. Scale bar = 400 μm. (E) Relative expression of CAF-related marker genes in PaSCs after culturing in parental PDLO conditioned media for 72 h. The mRNA expression was normalized to *GAPDH*, and expression is shown relative to the untreated conditions (-Dox). Mean, n=2. Supplemental Figure 4. (A) Scatter plot showing predicted interaction strengths of different cell types of PDAC samples from Peng et al.[33] and PDLOs. (B) Signaling patterns of selected pathways in different cell types of PDAC samples from Peng et al.^[33]^and PDLOs. (C) TNF signaling patterns in different cell types of PDAC samples from Peng et al.^[33]^and PDLOs. (D) Circle plot showing predicted interaction strengths of the ligand-receptor pair TNF-TNFRSF1B between different cell types of PDAC samples from Peng et al.^[33]^ and PDLOs. (F) GSEA plots showing enrichment of DEGs of PDLOs from different time points of the scRNAseq analysis compared to the -Dox control against the HALLMARK_TNF_SIGNALING_VIA_NFKB gene set. (F) Percentage of T cells migrated towards reduced T cell media with the addition of SSP1, PTN or, TNFa at indicated concentrations. Mean ± SD, n=2. (G) TNFα concentrations in parental PDLO-derived conditioned media from 5 independent pancreatic differentiation experiments (left) and relative levels normalized to the untreated -Dox control (right). Mean ± SD. Paired t-test ns, p>0.05. (H) Bar graph showing distribution of t-SNE clusters of murine fibroblasts, untreated or treated with respective ligands for 72 h, before being subjected to CyTOF analysis. (I) UMAP showing fibroblasts of a reanalyzed scRNA-seq data set from Carpenter et al.^20^. (J) Violin plot showing module score of CyTOF cluster 13 markers in fibroblasts of healthy and PDAC samples from from Carpenter et al.^20^ Unpaired t-test ****, p<0.0001, *, p<0.05, ns, p>0.05. (K) Expression of M1- and M2-polarisation marker genes relative to *HMBS* in macrophages. (L) Chemotactic migration of human macrophages towards PDLO-derived conditioned medium under the indicated conditions. C5a served as positive control. The cell count on the bottom side of the membrane (migrated cells), normalized to the initial top-side value (all cells after seeding), is plotted over time. Statistical significance was determined by ratio-paired t-test on the areas under the respective curves.* p < 0.05. Supplemental Figure 5. (A) Brightfield images of PDLO co-cultures used as input for scRNA-sequencing. Scale bar = 250 µm. (B) UMAP of cell types within the different co-culture conditions. (C) UMAP of cell-type-specific marker expression. (D) Cluster tree plot illustrating transcriptional similarity between PDLOs of time-series scRNA-seq data set (KRAS-PDLO_1), PDLOs of co-culture scRNA-seq data set (KRAS-PDLO_2) and Dox-treated parental PDLOs based on treatment conditions. (E) Heatmap showing log2FC values of selectively chosen ECM and inflammation-related genes from DEGs between KRAS^high^and KRAS^intermediate^clusters from both time-series and co-culture scRNA-seq data sets compared to parental PDLOs + Dox. (F) Cluster tree plot illustrating transcriptional similarity between PDLOs of time-series scRNA-seq data set (KRAS-PDLO_1) and PDLOs of co-culture scRNA-seq data set (KRAS-PDLO_2) based on KRAS clusters. (G) UMAP of the PDLO subset colored by KRAS groups and split by treatment condition (left) and co-culture condition (right). The samples from the monocultures were only included in one of two sequencing runs and therefore show overall lower cell numbers (see the Methods section for details). (H) Heatmap showing log2FC values of selectively chosen ECM and inflammation-related genes from DEGs between KRAS^high^and KRAS^intermediate^clusters compared to KRAS^low^ for the different co-culture conditions. (I) UMAP of the T cell subset split by the treatment condition. (J) UMAP of the T cell subset colored by Louvain clusters. (K) Dot plot of the T cell subset showing the expression of T cell-specific marker genes within the respective Louvain clusters. (L) UMAP of the T cell subset colored by the annotated CD4 and CD8 subpopulations. (M) Immunofluorescence images of PDLOs co-cultured with T cells for 72h. Scale bar = 50 µm. (N) Quantification of T cell subpopulations after direct co-culture with PDLOs assessed by flow cytometry. n = 3 T cells donors. Supplemental Figure 6. (A) UMAP of stellate cell subset split by the different treatment conditions. (B) UMAP of the stellate cell subset colored by the Louvain clusters. (C) Dot plot of the stellate cell subset grouped by the Louvain clusters showing the module scores for different CAF subpopulations using the top 100 DEGs from Toa et al[39] (D) Proportions of different CAF-subtypes within the stellate cells annotated by module scores using the top 100 DEGs from Toa et al.^[39]^ (E) Dot plot representing the average expression of canonical CAF marker in the different conditions. (F) Pathways from cell-cell communication analysis with a Euclidean distance greater than 10 based on their functional similarities.

## Data Availability

The sequencing data generated for this study have been deposited on Gene Expression Omnibus (GEO) database repository under the accession numbers GSE284392 (time-series scRNA-seq), GSE312209 (co-culture scRNA-seq), and GSE284390 (ATAC-seq).
