# Supplementary material for: An oncogenic KRAS-driven secretome involving TNFα promotes niche preparation prior to pancreatic cancer onset
Source: Mol Cancer. 2026 Feb 3;25:29. doi: 10.1186/s12943-025-02541-1 (PMC12903562; doi:10.1186/s12943-025-02541-1)
Supplement: Supplementary file 1 — Supplementary Material 1: Supplementary Figure 1. (A) Brightfield images of PDLOs ± Dox treatment. Scale bar = 250 µm. (B) Flow cytometry quantifying mCherry reporter expression after Dox treatment. (C) Dot plot showing expression of cell type markers in PDLO scRNA-seq data set. (D) KRAS copy numbers for hESC lines, PDAC lines and patient-derived organoids (PDOs) using probes targeting either the 3’ untranslated region (3’UTR) of the endogenous locus or exon 3, which detects the respective sequence in the endogenous locus and in the inserted piggyBac KRASG12D construct. Copy numbers are shown relative to the respective KRAS wild-type cell lines (parental hESCs, BxPC3 and PDO#38188). BU3 = monoallelic BU3 NGST-TetOn:KRASG12D line[92]. (E) Scatter plot showing correlation between KRASG12D expression and module scores of different pathways over the time of Dox treatment within the scRNA-seq data set. R = Pearson’s correlation coefficient. (F) Protein expression of phospho-ERK1/2 and ERK1/2 in PDLOs after indicated time-points of Dox treatment, in PDAC lines and PDOs. For quantification, phospho-ERK1/2 levels were normalized to ERK1/2 levels for each sample. n = 3 for KRAS-PDLOs, n = 2 for parental PDLOs and n = 1 for PDAC lines and PDOs. (G) Cell cycle analysis of PDLOs after indicated time-points of Dox treatment assessed by EdU quantification via flow cytometry. n = 4. (H) PCA plot demonstrating variance of chromatin accessibility between ATAC-seq samples. (I) Heatmap showing mean gene expression of TFs, overlapping in ATAC- and scRNA-seq. Supplemental Figure 2. (A) Proportion of the KRAS clusters within the individual time points of the scRNA-seq analysis. (B) Expression of the top 10 DEGs from each KRAS cluster compared to all other clusters. (C) Overlapping and unique DEGs of KRAShighand KRASintermediate compared to the KRASlow cluster with corresponding KEGG pathway analysis of unique DEGs. (D) Overall survival of TCGA-PAAD patients grouped based on KRAS cluster [file 12943_2025_2541_MOESM1_ESM.pdf]

Suppl. Fig. 1

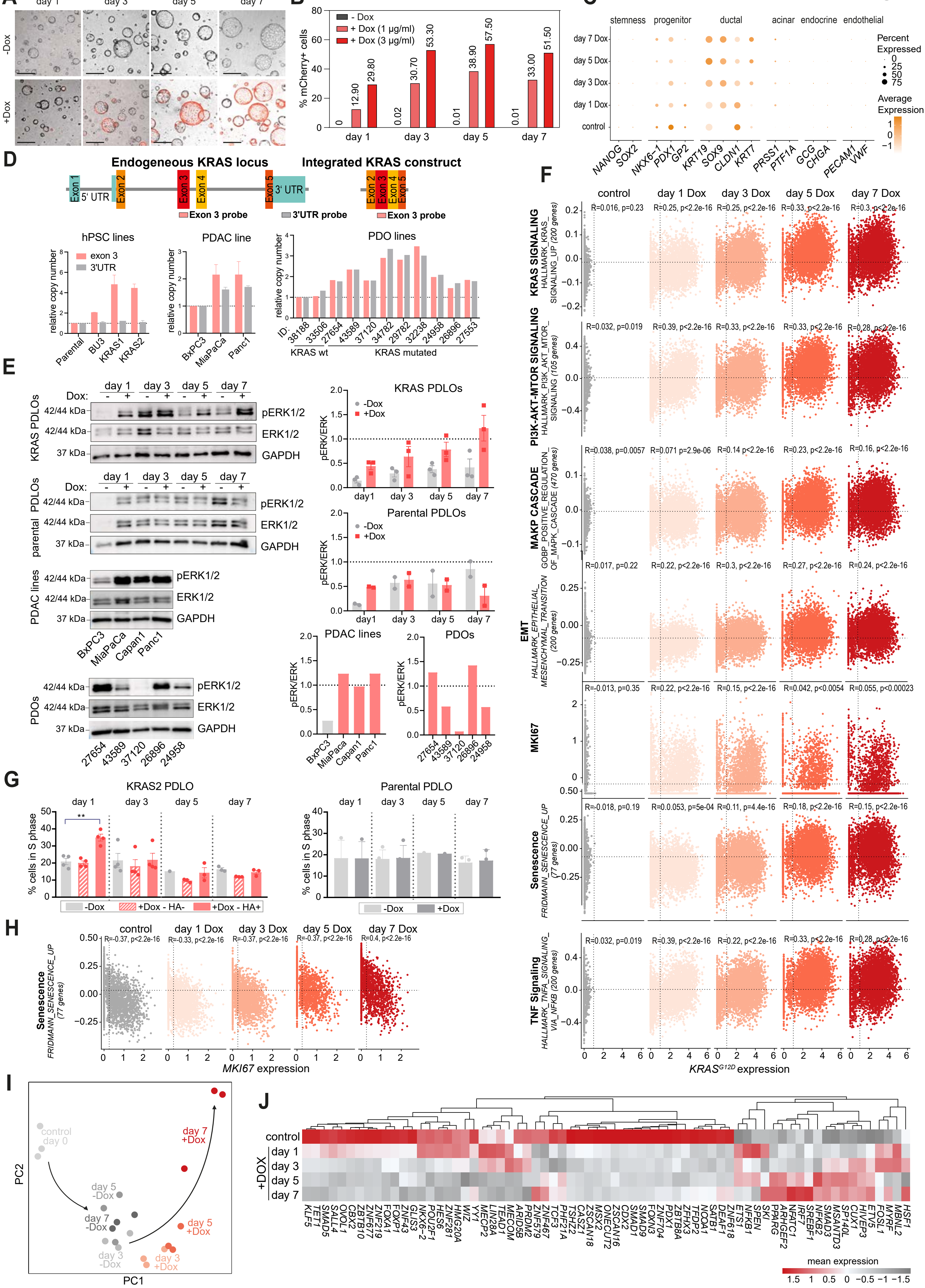

A
day 1
day 3
day 5
day 7
-Dox
+Dox
B
% mCherry+ cells
- Dox
+ Dox (1 µg/ml)
+ Dox (3 µg/ml)
0
12.90
29.80
0.02
30.70
53.30
0.01
38.90
57.50
0.01
33.00
51.50
day 1
day 3
day 5
day 7
C
stemness
progenitor
ductal
acinar
endocrine
endothelial
day 7 Dox
day 5 Dox
day 3 Dox
day 1 Dox
control
NANOG
SOX2
NKX6−1
PDX1
GP2
KRT19
SOX9
CLDN1
KRT7
PRSS1
PTF1A
GCG
CHGA
PECAM1
VWF
Percent Expressed
Average Expression
D
Endogeneous KRAS locus
Integrated KRAS construct
Exon 1
5' UTR
Exon 2
Exon 3
Exon 4
Exon 5
3' UTR
Exon 3 probe
3'UTR probe
hPSC lines
PDAC line
PDO lines
exon 3
3'UTR
relative copy number
Parental
BU3
KRAS1
KRAS2
BxPC3
MiaPaCa
Panc1
ID: 38188
33506
27654
43589
37120
34782
29782
32238
24958
26896
27553
KRAS wt
KRAS mutated
E
Dox:
day 1
day 3
day 5
day 7
KRAS PDLOs
parental PDLOs
PDAC lines
PDOs
42/44 kDa
37 kDa
pERK1/2
ERK1/2
GAPDH
BxPC3
MiaPaCa
Capan1
Panc1
27654
43589
37120
26896
24958
KRAS PDLOs
Parental PDLOs
PDAC lines
PDOs
pERK/ERK
-Dox
+Dox
F
control
day 1 Dox
day 3 Dox
day 5 Dox
day 7 Dox
KRAS SIGNALING
HALLMARK_KRAS_SIGNALING_UP (200 genes)
R=0.016, p=0.23
R=0.25, p<2.2e-16
R=0.25, p<2.2e-16
R=0.33, p<2.2e-16
R=0.3, p<2.2e-16
PI3K-AKT-MTOR SIGNALING
HALLMARK_PI3K_AKT_MTOR_SIGNALING (105 genes)
R=0.032, p=0.019
R=0.39, p<2.2e-16
R=0.33, p<2.2e-16
R=0.33, p<2.2e-16
R=0.28, p<2.2e-16
MAKP CASCADE
GOBP_POSITIVE_REGULATION_OF_MAPK_CASCADE (470 genes)
R=0.038, p=0.0057
R=0.071 p=2.9e-06
R=0.14 p<2.2e-16
R=0.23, p<2.2e-16
R=0.16, p<2.2e-16
EMT
HALLMARK_EPITHELIAL_MESENCHYMAL_TRANSITION (200 genes)
R=0.017, p=0.22
R=0.22, p<2.2e-16
R=0.3, p<2.2e-16
R=0.27, p<2.2e-16
R=0.24, p<2.2e-16
MKI67
R=-0.013, p=0.35
R=0.22, p<2.2e-16
R=0.15, p<2.2e-16
R=0.042, p<0.0054
R=0.055, p<0.00023
Senescence
FRIDMANN_SENESCENCE_UP (77 genes)
R=-0.018, p=0.19
R=0.053, p=5e-04
R=0.11, p=4.4e-16
R=0.18, p<2.2e-16
R=0.15, p<2.2e-16
TNF Signaling
HALLMARK_TNFA_SIGNALING_VIA_NFKB (200 genes)
R=0.032, p=0.019
R=0.39, p<2.2e-16
R=0.22, p<2.2e-16
R=0.33, p<2.2e-16
R=0.28, p<2.2e-16
KRAS^G12D expression
G
KRAS2 PDLO
Parental PDLO
% cells in S phase
-Dox
+Dox - HA-
+Dox - HA+
+Dox
H
Senescence
FRIDMANN_SENESCENCE_UP (77 genes)
R=-0.37, p<2.2e-16
R=-0.33, p<2.2e-16
R=-0.37, p<2.2e-16
R=-0.37, p<2.2e-16
R=0.4, p<2.2e-16
MKI67 expression
I
PC1
PC2
control day 0
day 5 -Dox
day 7 -Dox
day 3 -Dox
day 3 +Dox
day 5 +Dox
day 7 +Dox
J
control
+DOX
mean expression

Supplementary Figure 1

(**A**) Brightfield images of PDLOs ± Dox treatment. Scale bar = 250 µm.

(**B**) Flow cytometry quantifying mCherry reporter expression after Dox treatment.

(**C**) Dot plot showing expression of cell type markers in PDLO scRNA-seq data set.

(**D**) *KRAS* copy numbers for hESC lines, PDAC lines and patient-derived organoids (PDOs) using probes targeting either the 3' untranslated region (3'UTR) of the endogenous locus or exon 3, which detects the respective sequence in the endogenous locus and in the inserted piggyBac $KRAS^{G12D}$ construct. Copy numbers are shown relative to the respective KRAS wild-type cell lines (parental hESCs, BxPC3 and PDO#38188). BU3 = monoallelic BU3 NGST-TetOn:KRASG12D line[92].

(**E**) Protein expression of phospho-ERK1/2 and ERK1/2 in PDLOs after indicated time-points of Dox treatment, in PDAC lines and PDOs. For quantification, phospho-ERK1/2 levels were normalized to ERK1/2 levels for each sample. n = 3 for KRAS-PDLOs, n = 2 for parental PDLOs and n = 1 for PDAC lines and PDOs.

(**F**) Scatter plot showing correlation between $KRAS^{G12D}$ expression and module scores of different pathways over the time of Dox treatment within the scRNA-seq data set. R = Pearson's correlation coefficient.

(**G**) Cell cycle analysis of PDLOs after indicated time-points of Dox treatment assessed by EdU quantification via flow cytometry. n = 4.

(**H**) Scatter plot showing correlation between *MKI67* expression and module scores for senescence-related genes over the time of Dox treatment within the scRNA-seq data set. R = Pearson's correlation coefficient.

(**I**) PCA plot demonstrating variance of chromatin accessibility between ATAC-seq samples.

(**J**) Heatmap showing mean gene expression of TFs, overlapping in ATAC- and scRNA-seq.

Suppl. Fig. 2

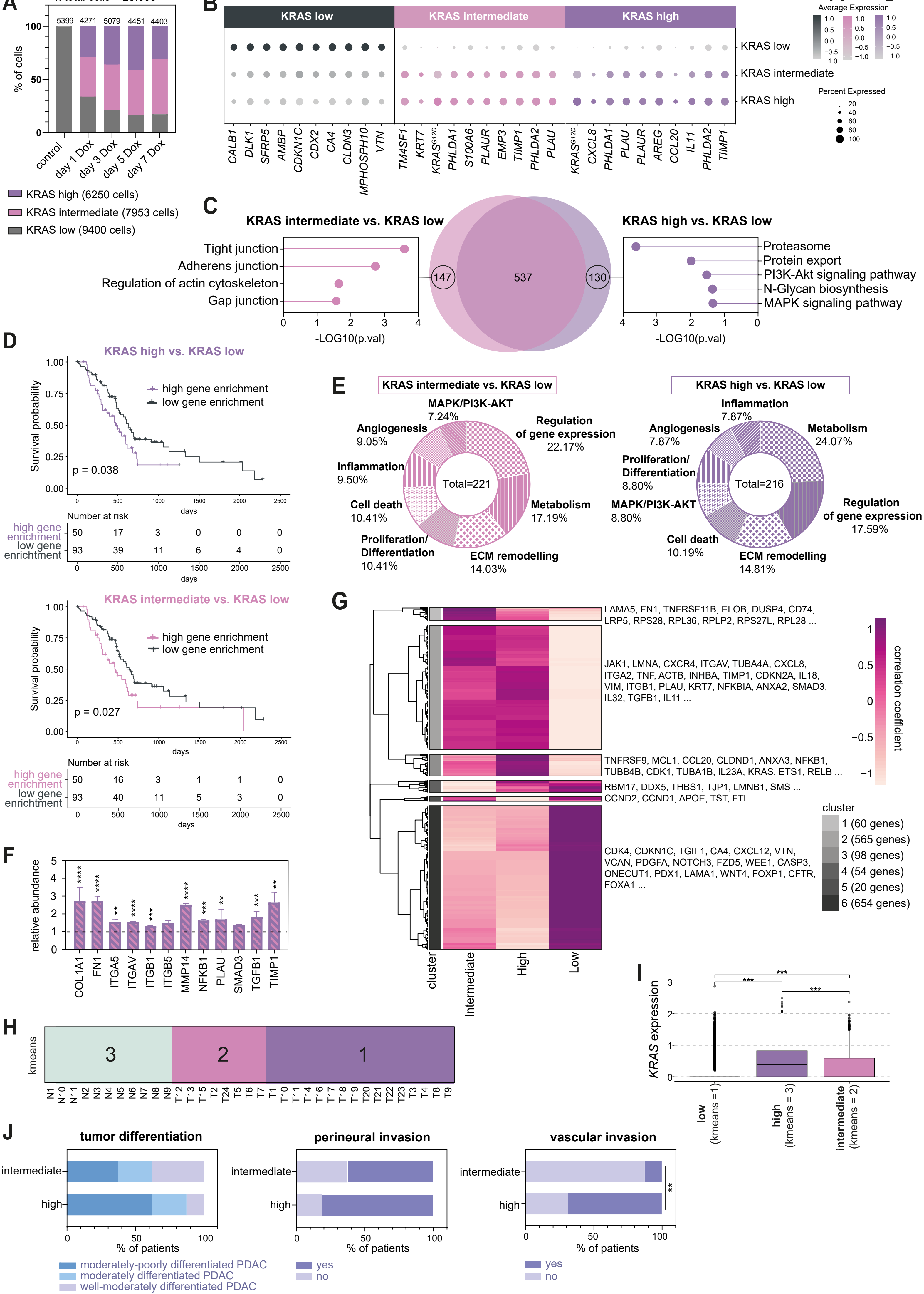
A
n total cells = 23.603
5399 4271 5079 4451 4403
% of cells
control
day 1 Dox
day 3 Dox
day 5 Dox
day 7 Dox
KRAS high (6250 cells)
KRAS intermediate (7953 cells)
KRAS low (9400 cells)
B
KRAS low
KRAS intermediate
KRAS high
Average Expression
Percent Expressed
CALB1 DLK1 SFRP5 AMBP CDKN1C CDX2 CA4 CLDN3 MPHOSPH10 VTN
TM4SF1 KRT7 KRAS G12D PHLDA1 S100A6 PLAUR EMP3 TIMP1 PHLDA2 PLAU
KRAS G12D CXCL8 PHLDA1 PLAU PLAUR AREG CCL20 IL11 PHLDA2 TIMP1
C
KRAS intermediate vs. KRAS low
Tight junction
Adherens junction
Regulation of actin cytoskeleton
Gap junction
-LOG10(p.val)
147
537
130
KRAS high vs. KRAS low
Proteasome
Protein export
PI3K-Akt signaling pathway
N-Glycan biosynthesis
MAPK signaling pathway
-LOG10(p.val)
D
KRAS high vs. KRAS low
Survival probability
high gene enrichment
low gene enrichment
p = 0.038
days
Number at risk
high gene enrichment 50 17 3 0 0 0
low gene enrichment 93 39 11 6 4 0
KRAS intermediate vs. KRAS low
high gene enrichment
low gene enrichment
p = 0.027
Number at risk
high gene enrichment 50 16 3 1 1 0
low gene enrichment 93 40 11 5 3 0
E
KRAS intermediate vs. KRAS low
MAPK/PI3K-AKT 7.24%
Regulation of gene expression 22.17%
Angiogenesis 9.05%
Inflammation 9.50%
Total=221
Metabolism 17.19%
Cell death 10.41%
Proliferation/ Differentiation 10.41%
ECM remodelling 14.03%
KRAS high vs. KRAS low
Inflammation 7.87%
Angiogenesis 7.87%
Metabolism 24.07%
Proliferation/ Differentiation 8.80%
Total=216
MAPK/PI3K-AKT 8.80%
Regulation of gene expression 17.59%
Cell death 10.19%
ECM remodelling 14.81%
F
relative abundance
COL1A1 FN1 ITGA5 ITGAV ITGB1 ITGB5 MMP14 NFKB1 PLAU SMAD3 TGFB1 TIMP1
G
LAMA5, FN1, TNFRSF11B, ELOB, DUSP4, CD74, LRP5, RPS28, RPL36, RPLP2, RPS27L, RPL28 ...
JAK1, LMNA, CXCR4, ITGAV, TUBA4A, CXCL8, ITGA2, TNF, ACTB, INHBA, TIMP1, CDKN2A, IL18, VIM, ITGB1, PLAU, KRT7, NFKBIA, ANXA2, SMAD3, IL32, TGFB1, IL11 ...
TNFRSF9, MCL1, CCL20, CLDND1, ANXA3, NFKB1, TUBB4B, CDK1, TUBA1B, IL23A, KRAS, ETS1, RELB ...
RBM17, DDX5, THBS1, TJP1, LMNB1, SMS ...
CCND2, CCND1, APOE, TST, FTL ...
CDK4, CDKN1C, TGIF1, CA4, CXCL12, VTN, VCAN, PDGFA, NOTCH3, FZD5, WEE1, CASP3, ONECUT1, PDX1, LAMA1, WNT4, FOXP1, CFTR, FOXA1 ...
correlation coefficient
cluster
1 (60 genes)
2 (565 genes)
3 (98 genes)
4 (54 genes)
5 (20 genes)
6 (654 genes)
cluster
Intermediate
High
Low
H
kmeans
3
2
1
N1 N10 N11 N2 N3 N4 N5 N6 N7 N8 N9 T12 T13 T15 T2 T24 T5 T6 T7 T1 T10 T11 T14 T16 T17 T18 T19 T20 T21 T22 T23 T3 T4 T8 T9
I
KRAS expression
low (kmeans =1)
high (kmeans = 3)
intermediate (kmeans = 2)
J
tumor differentiation
intermediate
high
% of patients
moderately-poorly differentiated PDAC
moderately differentiated PDAC
well-moderately differentiated PDAC
perineural invasion
intermediate
high
% of patients
yes
no
vascular invasion
intermediate
high
% of patients
yes
no

Supplemental Figure 2

(**A**) Proportion of the KRAS clusters within the individual time points of the scRNA-seq analysis.

(**B**) Expression of the top 10 DEGs from each KRAS cluster compared to all other clusters.

(**C**) Overlapping and unique DEGs of $KRAS^{high}$ and $KRAS^{intermediate}$ compared to the $KRAS^{low}$ cluster with corresponding KEGG pathway analysis of unique DEGs.

(**D**) Overall survival of TCGA-PAAD patients grouped based on KRAS cluster DEGs by gene set variance analysis (GSVA).

(**E**) Pie charts showing the distribution of grouped GO terms from DEGs of $KRAS^{high}$ and $KRAS^{intermediate}$ compared to $KRAS^{low}$.

(**F**) Bar plot showing the relative abundance of proteins after 7 days of Dox treatment. Mean ± SD, n=1 in triplicates. Unpaired t-test **, p<0.01, ***, p<0.001; ****, p<0.0001.

(**G**) Heatmap showing correlation coefficients of KRAS deep learning classifiers.

(**H**) K-means grouping of healthy (N) and PDAC samples (T) from Peng et al.[33] based on KRAS deep learning classifiers.

(**I**) Box plot showing *KRAS* expression of PDAC samples from Peng et al.[33]grouped by k-means based on KRAS deep learning classifiers. Wilcoxon test ***, p<0.0001.

(**J**) Bar plots showing tumor differentiation (left), perineural invasion (middle), and vascular invasion (right) of PDAC samples from Peng et al. [33] grouped by k-means based on KRAS deep learning classifiers. Chi-square test **, p<0.01.

Suppl. Fig. 3

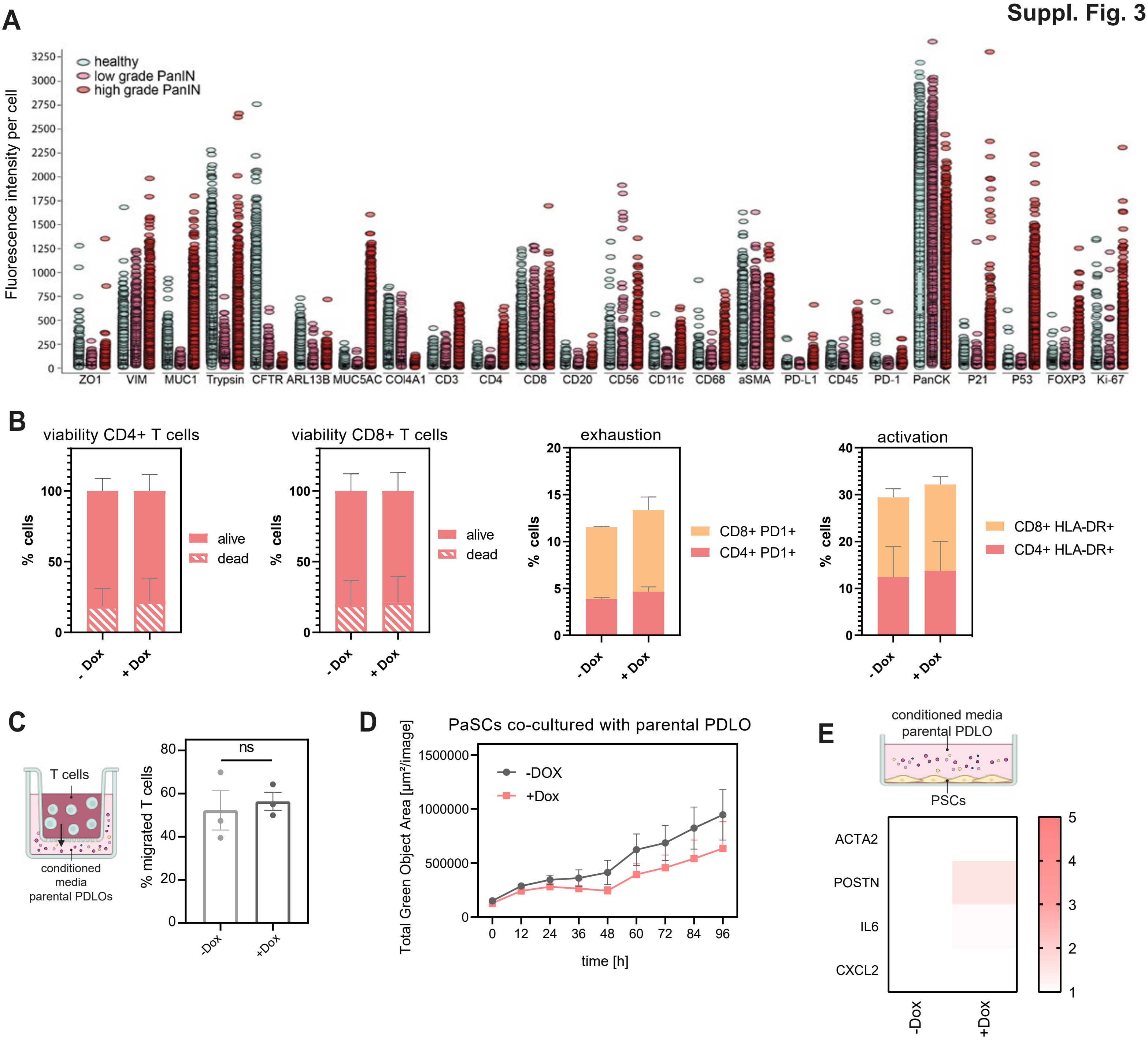
A
healthy
low grade PanIN
high grade PanIN
Fluorescence intensity per cell
ZO1
VIM
MUC1
Trypsin
CFTR
ARL13B
MUC5AC
COl4A1
CD3
CD4
CD8
CD20
CD56
CD11c
CD68
aSMA
PD-L1
CD45
PD-1
PanCK
P21
P53
FOXP3
Ki-67
B
viability CD4+ T cells
viability CD8+ T cells
exhaustion
activation
% cells
alive
dead
CD8+ PD1+
CD4+ PD1+
CD8+ HLA-DR+
CD4+ HLA-DR+
- Dox
+ Dox
C
T cells
conditioned media parental PDLOs
% migrated T cells
ns
-Dox
+Dox
D
PaSCs co-cultured with parental PDLO
Total Green Object Area [µm²/image]
-DOX
+Dox
time [h]
E
conditioned media parental PDLO
PSCs
ACTA2
POSTN
IL6
CXCL2
-Dox
+Dox

Supplemental Figure 3

(**A**) Fluorescence intensities of all markers after initial background subtraction on human PanIN lesions subjected to 24-plex immunofluorescence staining.

(**B**) Percentage of viable, exhausted, and activated T cells after culturing in PDLO conditioned media for 72 h determined by flow cytometry. Mean ± SD, n=2.

(**C**) Percentage of T cells migrating towards PDLO conditioned media derived from a control line without $KRAS^{G12D}$ construct (parental PDLOs). Mean ± SEM of 3 independent experiments. Unpaired t-test ns, p>0.05.

(**D**) Representative images of parental PDLOs co-cultured with GFP-labeled pancreatic stellate cells (PaSCs) and respective quantification of the total green area over time of live-cell imaging. Mean ± SEM, n=3. Unpaired t-test ***, p<0.001; ****, p<0.0001. Scale bar = 400 µm.

(**E**) Relative expression of CAF-related marker genes in PaSCs after culturing in parental PDLO conditioned media for 72 h. The mRNA expression was normalized to *GAPDH*, and expression is shown relative to the untreated conditions (-Dox). Mean, n=2

Suppl. Fig. 4

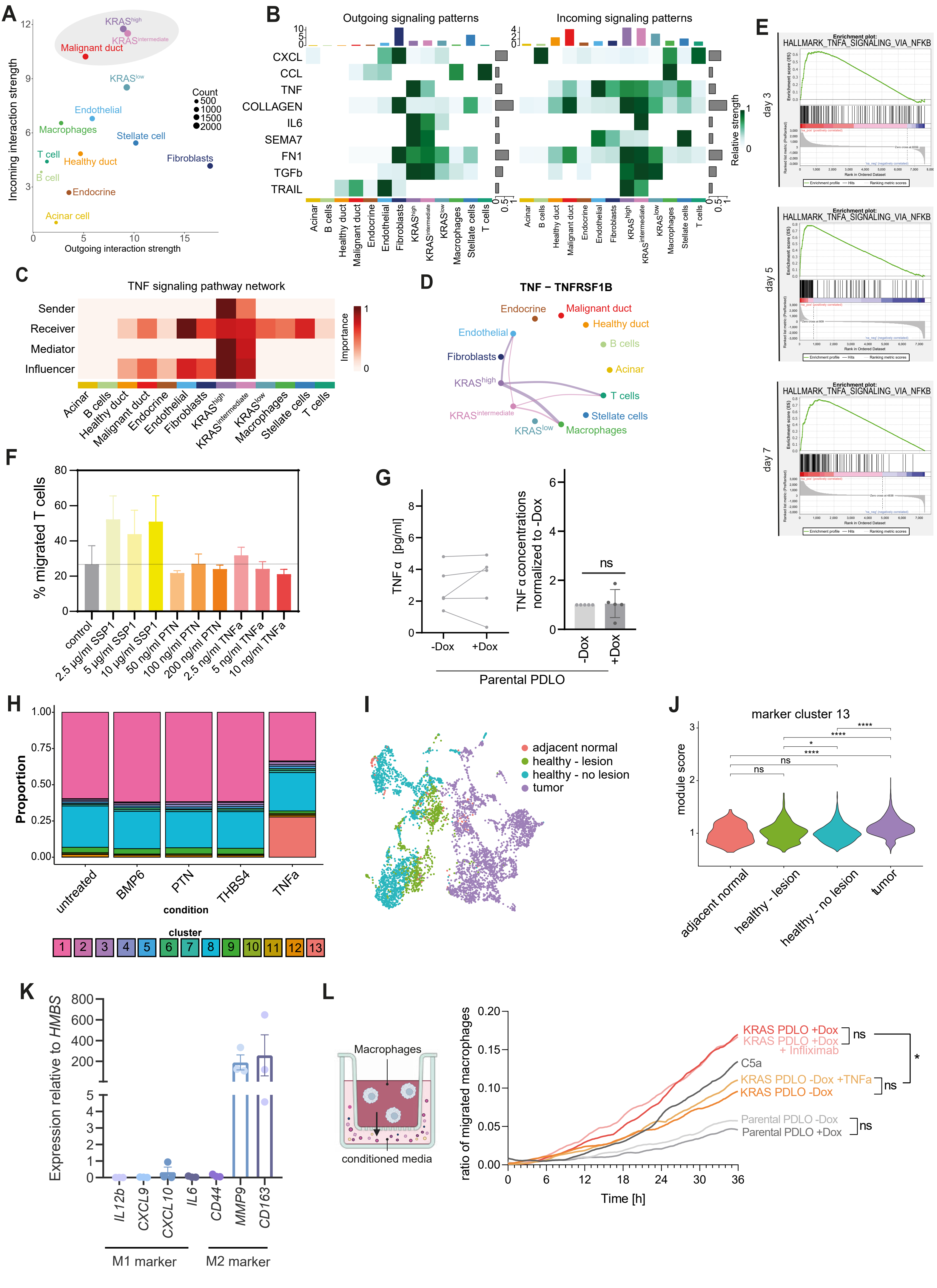

A
Incoming interaction strength
Outgoing interaction strength
Count
500
1000
1500
2000
KRAS high
KRAS intermediate
Malignant duct
KRAS low
Endothelial
Macrophages
Stellate cell
T cell
Healthy duct
Fibroblasts
B cell
Endocrine
Acinar cell
B
Outgoing signaling patterns
Incoming signaling patterns
CXCL
CCL
TNF
COLLAGEN
IL6
SEMA7
FN1
TGFb
TRAIL
Relative strength
Acinar
B cells
Healthy duct
Malignant duct
Endocrine
Endothelial
Fibroblasts
Macrophages
Stellate cells
T cells
C
TNF signaling pathway network
Sender
Receiver
Mediator
Influencer
Importance
D
TNF – TNFRSF1B
E
HALLMARK_TNFA_SIGNALING_VIA_NFKB
day 3
day 5
day 7
F
% migrated T cells
control
2.5 µg/ml SSP1
5 µg/ml SSP1
10 µg/ml SSP1
50 ng/ml PTN
100 ng/ml PTN
200 ng/ml PTN
2.5 ng/ml TNFa
5 ng/ml TNFa
10 ng/ml TNFa
G
TNF α [pg/ml]
TNF α concentrations normalized to -Dox
ns
-Dox
+Dox
Parental PDLO
H
Proportion
untreated
BMP6
PTN
THBS4
TNFa
condition
cluster
I
adjacent normal
healthy - lesion
healthy - no lesion
tumor
J
marker cluster 13
module score
ns
*
****
adjacent normal
healthy - lesion
healthy - no lesion
tumor
K
Expression relative to HMBS
IL12b
CXCL9
CXCL10
IL6
CD44
MMP9
CD163
M1 marker
M2 marker
L
Macrophages
conditioned media
ratio of migrated macrophages
Time [h]
KRAS PDLO +Dox
KRAS PDLO +Dox + Infliximab
C5a
KRAS PDLO -Dox +TNFa
KRAS PDLO -Dox
Parental PDLO -Dox
Parental PDLO +Dox

Supplemental Figure 4

(**A**) Scatter plot showing predicted interaction strengths of different cell types of PDAC samples from Peng et al.[33] and PDLOs.

(**B**) Signaling patterns of selected pathways in different cell types of PDAC samples from Peng et al.[33]and PDLOs.

(**C**) TNF signaling patterns in different cell types of PDAC samples from Peng et al.[33]and PDLOs.

(**D**) Circle plot showing predicted interaction strengths of the ligand-receptor pair TNF-TNFRSF1B between different cell types of PDAC samples from Peng et al.[33] and PDLOs.

(**F**) GSEA plots showing enrichment of DEGs of PDLOs from different time points of the scRNAseq analysis compared to the -Dox control against the HALLMARK_TNF_SIGNALING_VIA_NFKB gene set.

(**F**) Percentage of T cells migrated towards reduced T cell media with the addition of SSP1, PTN or, TNFa at indicated concentrations. Mean ± SD, n=2.

(**G**) TNFα concentrations in parental PDLO-derived conditioned media from 5 independent pancreatic differentiation experiments (left) and relative levels normalized to the untreated -Dox control (right). Mean ± SD. Paired t-test ns, p>0.05

(**H**) Bar graph showing distribution of t-SNE clusters of murine fibroblasts, untreated or treated with respective ligands for 72 h, before being subjected to CyTOF analysis.

(**I**) UMAP showing fibroblasts of a reanalyzed scRNA-seq data set from Carpenter et al.[20].

(**J**) Violin plot showing module score of CyTOF cluster 13 markers in fibroblasts of healthy and PDAC samples from from Carpenter et al.[20] Unpaired t-test ****, p<0.0001, *, p<0.05, ns, p>0.05

(**K**) Expression of M1- and M2-polarisation marker genes relative to *HMBS* in macrophages.

(**L**) Chemotactic migration of human macrophages towards PDLO-derived conditioned medium under the indicated conditions. C5a served as positive control. The cell count on the bottom side of the membrane (migrated cells), normalized to the initial top-side value (all cells after seeding), is plotted over time. Statistical significance was determined by ratio-paired t-test on the areas under the respective curves.* p < 0.05.

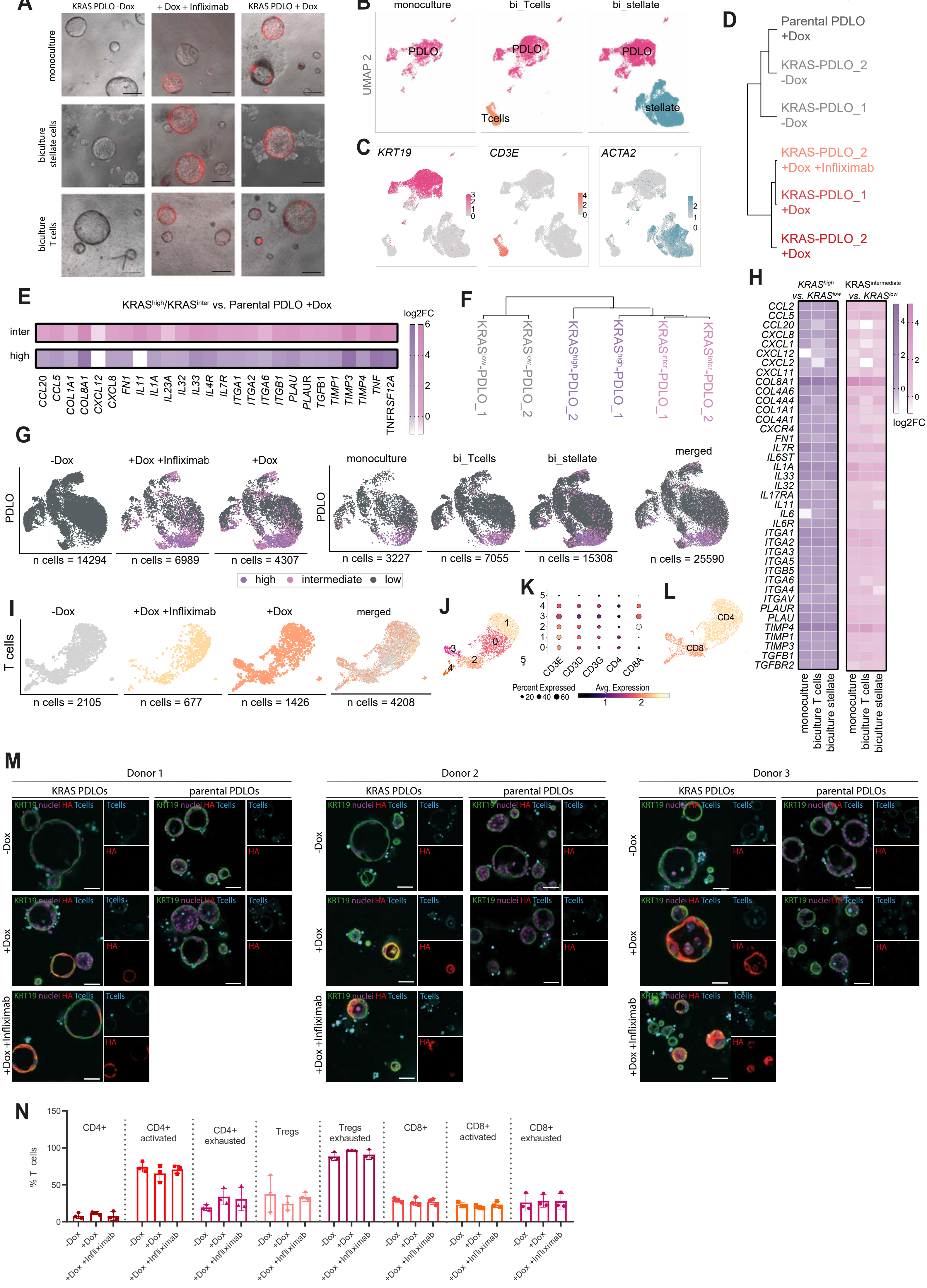
Supplementary Figure 5
A
KRAS PDLO -Dox
KRAS PDLO + Dox + Infliximab
KRAS PDLO + Dox
monoculture
biculture stellate cells
biculture T cells
B
monoculture
bi_Tcells
bi_stellate
UMAP 2
PDLO
Tcells
stellate
C
KRT19
CD3E
ACTA2
D
Parental PDLO +Dox
KRAS-PDLO_2 -Dox
KRAS-PDLO_1 -Dox
KRAS-PDLO_2 +Dox +Infliximab
KRAS-PDLO_1 +Dox
KRAS-PDLO_2 +Dox
E
KRAS^high/KRAS^inter vs. Parental PDLO +Dox
inter
high
log2FC
CCL20 CCL5 COL1A1 COL8A1 CXCL12 CXCL8 FN1 IL11 IL1A IL23A IL32 IL33 IL4R IL7R ITGA1 ITGA2 ITGA6 ITGB1 PLAU PLAUR TGFB1 TIMP1 TIMP3 TIMP4 TNF TNFRSF12A
F
KRAS^low-PDLO_1
KRAS^low-PDLO_2
KRAS^high-PDLO_2
KRAS^high-PDLO_1
KRAS^inter-PDLO_1
KRAS^inter-PDLO_2
G
-Dox
+Dox +Infliximab
+Dox
monoculture
bi_Tcells
bi_stellate
merged
PDLO
n cells = 14294
n cells = 6989
n cells = 4307
n cells = 3227
n cells = 7055
n cells = 15308
n cells = 25590
high intermediate low
H
KRAS^high vs. KRAS^low
KRAS^intermediate vs. KRAS^low
CCL2 CCL5 CCL20 CXCL8 CXCL1 CXCL12 CXCL2 CXCL11 COL8A1 COL4A6 COL4A4 COL1A1 COL4A1 CXCR4 FN1 IL7R IL6ST IL1A IL33 IL32 IL17RA IL11 IL6 IL6R ITGA1 ITGA2 ITGA3 ITGA5 ITGB5 ITGA6 ITGA4 ITGAV PLAUR PLAU TIMP4 TIMP1 TIMP3 TGFB1 TGFBR2
log2FC
monoculture
biculture T cells
biculture stellate
I
T cells
-Dox
+Dox +Infliximab
+Dox
merged
n cells = 2105
n cells = 677
n cells = 1426
n cells = 4208
J
K
CD3E CD3D CD3G CD4 CD8A
Percent Expressed
20 40 60
Avg. Expression
L
CD4
CD8
M
Donor 1
Donor 2
Donor 3
KRAS PDLOs
parental PDLOs
KRT19 nuclei HA Tcells
Tcells
HA
-Dox
+Dox
+Dox +Infliximab
N
% T cells
CD4+
CD4+ activated
CD4+ exhausted
Tregs
Tregs exhausted
CD8+
CD8+ activated
CD8+ exhausted
-Dox
+Dox
+Dox +Infliximab

Supplemental Figure 5

(**A**) Brightfield images of PDLO co-cultures used as input for scRNA-sequencing. Scale bar = 250 µm.

(**B**) UMAP of cell types within the different co-culture conditions.

(**C**) UMAP of cell-type-specific marker expression.

(**D**) Cluster tree plot illustrating transcriptional similarity between PDLOs of time-series scRNA-seq data set (KRAS-PDLO_1), PDLOs of co-culture scRNA-seq data set (KRAS-PDLO_2) and Dox-treated parental PDLOs based on treatment conditions.

(**E**) Heatmap showing log2FC values of selectively chosen ECM and inflammation-related genes from DEGs between $KRAS^{high}$ and $KRAS^{intermediate}$ clusters from both time-series and co-culture scRNA-seq data sets compared to parental PDLOs + Dox.

(**F**) Cluster tree plot illustrating transcriptional similarity between PDLOs of time-series scRNA-seq data set (KRAS-PDLO_1) and PDLOs of co-culture scRNA-seq data set (KRAS-PDLO_2) based on KRAS clusters.

(**G**) UMAP of the PDLO subset colored by KRAS groups and split by treatment condition (left) and co-culture condition (right). The samples from the monocultures were only included in one of two sequencing runs and therefore show overall lower cell numbers (see the Methods section for details).

(**H**) Heatmap showing log2FC values of selectively chosen ECM and inflammation-related genes from DEGs between $KRAS^{high}$ and $KRAS^{intermediate}$ clusters compared to $KRAS^{low}$ for the different co-culture conditions.

(**I**) UMAP of the T cell subset split by the treatment condition.

(**J**) UMAP of the T cell subset colored by Louvain clusters.

(**K**) Dot plot of the T cell subset showing the expression of T cell-specific marker genes within the respective Louvain clusters.

(**L**) UMAP of the T cell subset colored by the annotated CD4 and CD8 subpopulations.

(**M**) Immunofluorescence images of PDLOs co-cultured with T cells for 72h. Scale bar = 50 µm.

(**N**) Quantification of T cell subpopulations after direct co-culture with PDLOs assessed by flow cytometry. n = 3 T cells donors.

Supplementary Figure 6

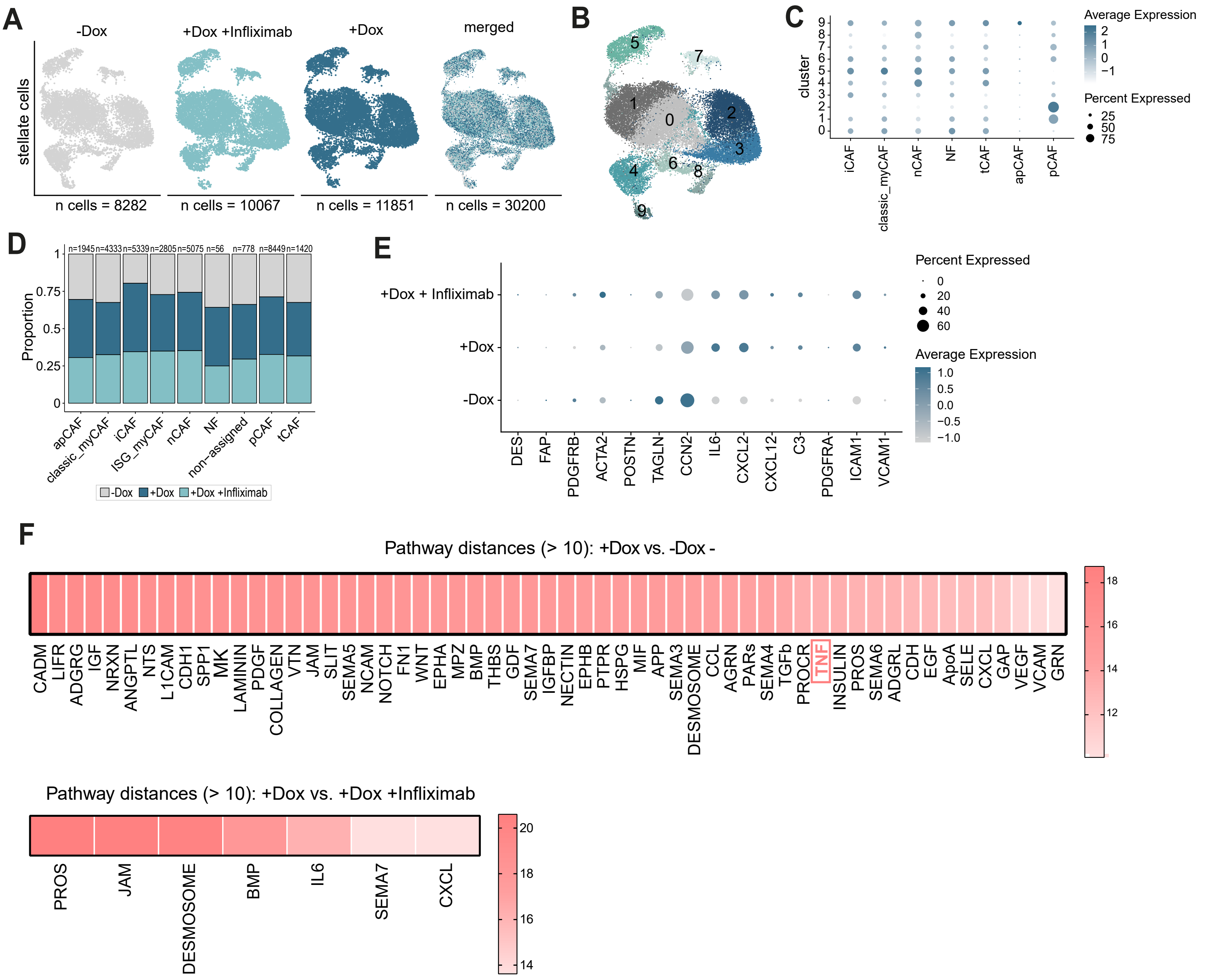

Supplemental Figure 6

(**A**) UMAP of stellate cell subset split by the different treatment conditions.

(**B**) UMAP of the stellate cell subset colored by the Louvain clusters.

(**C**) Dot plot of the stellate cell subset grouped by the Louvain clusters showing the module scores for different CAF subpopulations using the top 100 DEGs from Toa et al[39]

(**D**) Proportions of different CAF-subtypes within the stellate cells annotated by module scores using the top 100 DEGs from Toa et al.[39]

(**E**) Dot plot representing the average expression of canonical CAF marker in the different conditions.

(**F**) Pathways from cell-cell communication analysis with a Euclidean distance greater than 10 based on their functional similarities.
